# Development and validation of polygenic risk scores for prediction of breast cancer and breast cancer subtypes in Chinese women

**DOI:** 10.1186/s12885-022-09425-3

**Published:** 2022-04-08

**Authors:** Can Hou, Bin Xu, Yu Hao, Daowen Yang, Huan Song, Jiayuan Li

**Affiliations:** 1 West China Biomedical Big Data Center, West China Hospital, Sichuan University, No. 37 Guo Xue Xiang, Chengdu, 610047 Sichuan China; 2 Department of Epidemiology and Biostatistics, West China School of Public Health and West China Fourth Hospital, Sichuan University, No.16 Ren Min Nan Lu, Chengdu, 610041 Sichuan China; 3 Med-X Center for Informatics, Sichuan University, Chengdu, China; 4 Robot Perception and Control Joint Lab, Sichuan University & Aisono, Chengdu, China

**Keywords:** Breast cancer, Polygenic risk score, Single nucleotide polymorphisms, Artificial neural network, Estrogen receptor-negative breast cancer

## Abstract

**Background:**

Studies investigating breast cancer polygenic risk score (PRS) in Chinese women are scarce. The objectives of this study were to develop and validate PRSs that could be used to stratify risk for overall and subtype-specific breast cancer in Chinese women, and to evaluate the performance of a newly proposed Artificial Neural Network (ANN) based approach for PRS construction.

**Methods:**

The PRSs were constructed using the dataset from a genome-wide association study (GWAS) and validated in an independent case-control study. Three approaches, including repeated logistic regression (RLR), logistic ridge regression (LRR) and ANN based approach, were used to build the PRSs for overall and subtype-specific breast cancer based on 24 selected single nucleotide polymorphisms (SNPs). Predictive performance and calibration of the PRSs were evaluated unadjusted and adjusted for Gail-2 model 5-year risk or classical breast cancer risk factors.

**Results:**

The primary PRS_ANN_ and PRS_LRR_ both showed modest predictive ability for overall breast cancer (odds ratio per interquartile range increase of the PRS in controls [IQ-OR] 1.76 vs 1.58; area under the receiver operator characteristic curve [AUC] 0.601 vs 0.598) and remained to be predictive after adjustment. Although estrogen receptor negative (ER^−^) breast cancer was poorly predicted by the primary PRSs, the ER^−^ PRSs trained solely on ER^−^ breast cancer cases saw a substantial improvement in predictions of ER^−^ breast cancer.

**Conclusions:**

The 24 SNPs based PRSs can provide additional risk information to help breast cancer risk stratification in the general population of China. The newly proposed ANN approach for PRS construction has potential to replace the traditional approaches, but more studies are needed to validate and investigate its performance.

**Supplementary Information:**

The online version contains supplementary material available at 10.1186/s12885-022-09425-3.

## Background

Breast cancer is the most common type of malignant neoplasm and the second leading cause of cancer deaths in women worldwide [1]. The Global Burden of Disease (GBD) Study estimated that in 2017, breast cancer lead to over 17 million Disability-Adjusted Life Years (DALYs) and 600,000 deaths around the world [2]. Although the incidence of breast cancer is much lower in China than in the United States and European countries, the surge in the incidence in the largest population in the world over the past few decades has made breast cancer a major public health issue that seriously endangers the health of women in China [3].

The etiology of breast cancer is multifactorial, with both non-genetic risk factors (including reproductive factors, exogenous hormonal medication, and lifestyle factors) and inherited genetic risk factors playing important roles [4–8]. Multiple pathogenic variants of the *BRCA1* and *BRCA2* genes that confer high relative risks of breast cancer have been identified [9]. However, these variants are too rare in the general population to explain more than a small proportion of breast cancer cases [10, 11], especially among Chinese women where the prevalence of *BRCA1* and *BRCA2* mutations is lower than that in women of European ancestry [12]. In addition to these highly penetrant rare variants, more than 180 common single nucleotide polymorphisms (SNPs) that are associated with breast cancer risk have been identified in genome-wide association studies (GWASs) [13]. Each of these SNPs confers only a small risk of developing breast cancer, but when summarized in the form of a polygenic risk score (PRS), their combined effect can be substantial [14].

Breast cancer PRSs have been shown to have sufficient predictive power to aid risk stratification, and some have already been implemented in clinical practice [15, 16]. However, there is a lack of studies examining PRSs in Chinese women, since the majority of GWASs and other studies of breast cancer PRSs conducted to date were conducted among women of European ancestry [13]. Among the limited studies investigating breast cancer PRSs in Chinese women [17–21], the biggest limitation is the lack of validation using independent datasets. These studies used the same datasets to estimate the PRS weighting parameters and to evaluate the PRSs, which limited the value of the results as a true reflection of the performance of the PRSs. Furthermore, as highlighted by some recent studies, more efforts are needed to optimize PRSs for the prediction of estrogen receptor (ER) negative (ER^−^) breast cancer [22, 23], which is more aggressive and less common than estrogen receptor positive (ER^+^) breast cancer. Better prediction of ER-specific breast cancer could enable selection of high-risk women who might benefit from prevention with endocrine therapies.

The primary aim of this study was to develop and validate PRSs for use in stratification of the risk of breast cancer and subtype-specific breast cancer in Chinese women. To that end, we used a GWAS dataset to develop PRSs and validated them in an independent test set from a case-control study. We also aimed to compare different approaches for calculating PRSs, including a newly proposed artificial neural network (ANN)-based approach.

## Methods

### Study design and participants

**Fig. 1:**
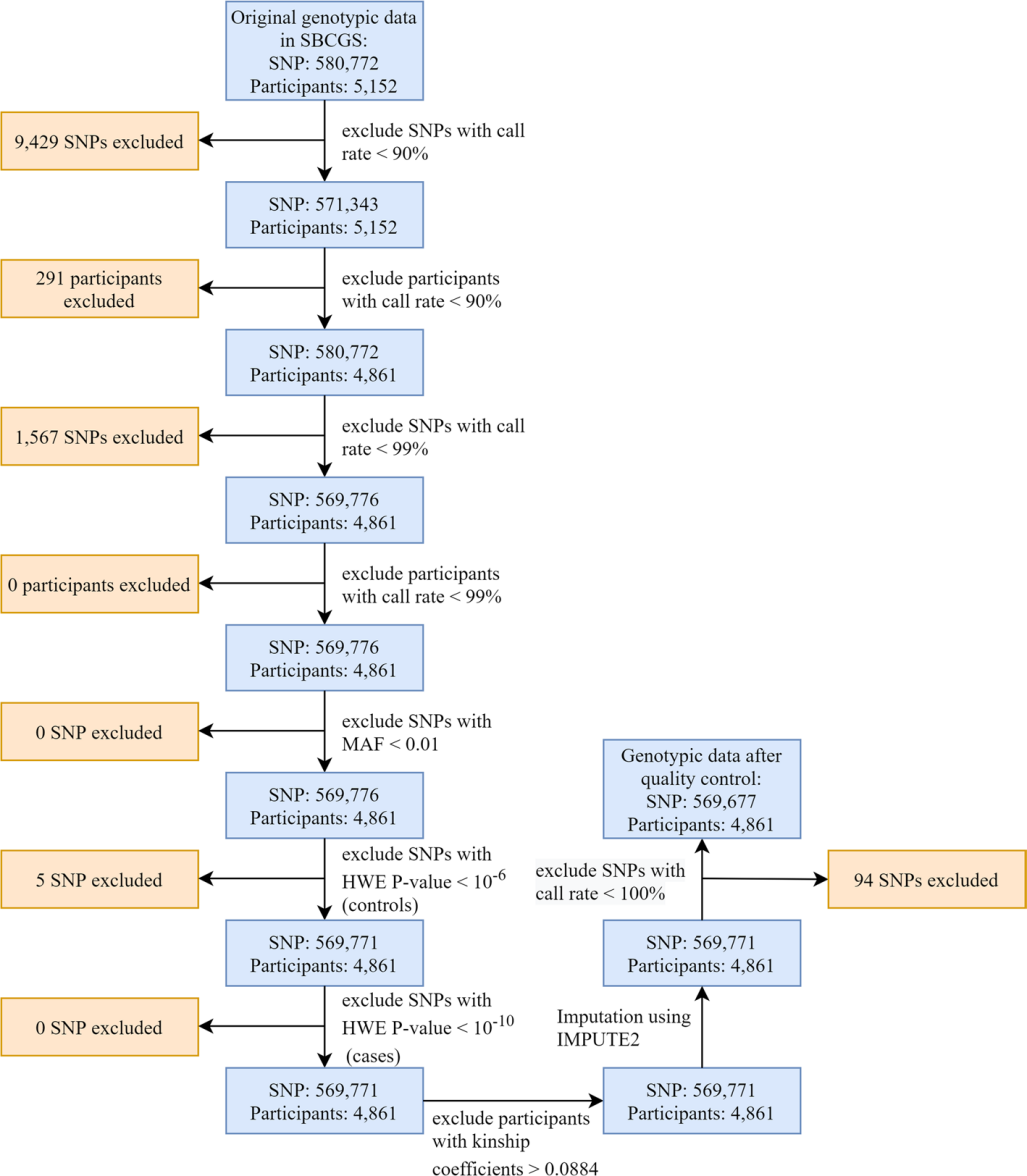
Flowchart of the quality control process of the genotypic data in SBCGS. Quality control procedures were carried out using PLINK 1.9. HWE: Hardy–Weinberg equilibrium; MAF: minor allele frequency; SBCGS: Shanghai Breast Cancer Genetics Study; SNPs: single nucleotide polymorphisms

The dataset used for PRS development was obtained from the Shanghai Breast Cancer Genetics Study (SBCGS) [24]. The SBCGS was conducted in 5152 participants (2867 case participants and 2285 control participants) from the following four population-based studies conducted among Chinese women in urban Shanghai between 1996 and 2005: the Shanghai Breast Cancer Study [25], the Shanghai Breast Cancer Survival Study [26], the Shanghai Endometrial Cancer Study (contributing controls only) [27] and the Shanghai Women’s Health Study [28]. The samples from the SBCGS were genotyped using Affymetrix Genome-Wide Human SNP Array 6.0. The raw individual-level genotype dataset was provided by the Database of Genotypes and Phenotypes (dbGaP) project phs000799.v1.p1 (https://www.ncbi.nlm.nih.gov/gap). The quality control (QC) procedures applied to the SBCGS dataset are described in Fig. 1. Briefly, we excluded SNPs and samples with a call rate < 99%. We also excluded SNPs with a minor allele frequency < 1%, SNPs with Hardy–Weinberg equilibrium (HWE) test *P* < 10^− 6^ and *P* < 10^− 10^ for controls and cases, respectively, and samples with KING-robust kinship coefficients > 0.0884 (second-degree relations, first-degree relations and duplicate samples) [29]. QC and imputation were performed using PLINK 1.9 and IMPUTE2 software [30, 31]. After QC procedures, the final dataset consisted of 4861 participants (2722 case participants and 2139 control participants) and 569,677 SNPs.

The independent test set used for PRS validation was obtained from the Sichuan Breast Cancer Case-Control Study (SBCCS) conducted in Chengdu, Sichuan Province. The study design has been described in detail elsewhere [6]. In brief, the SBCCS was conducted in 794 case participants and 805 control participants between 2014 and 2015. Case participants were recruited from primary breast cancer patients diagnosed in three government-owned hospitals, whereas control participants were recruited from healthy women undergoing annual physical examination in two physical examination centers. A standardized questionnaire was used to collect demographic and breast cancer risk factor information from participants. Clinical characteristics of case participants were directly exported from hospitals’ information systems. Blood samples were collected from all participants on the day of the questionnaire survey and stored at − 80 °C prior to DNA extraction. DNA was extracted from blood samples using whole blood genomic DNA extraction kits (Tiangen Biotech Company, Beijing, China) and stored at − 80 °C. In the current study, we included 826 DNA samples from 376 control participants and 431 case participants that were available in 2019.

### SNP selection and genotyping

We generated two sets of SNPs as potential candidates for genotyping in the SBCCS. The first set of SNPs was selected by reviewing association studies or meta-analyses. Due to budget limitations and the“diminishing returns” effect [13], we focused on susceptible SNPs that were identified in previous smaller studies and selected 28 SNPs that had been widely found to be associated with breast cancer risk in the Chinese population (Table S1). Thirteen SNPs were not represented in the SBCGS dataset, among which five SNPs (rs1801133, rs4973768, rs854560, rs1695 and rs9282861) were excluded because their eligible proxy SNPs, defined as linkage disequilibrium (LD) measure *R*^2^ > 0.9 determined using the LDLink tool [32], were also not represented in the SBCGS dataset. The remaining eight SNPs were replaced by corresponding proxies (rs1137101 replaced by rs10789190; rs10941679 replaced by rs4479849; rs662 replaced by rs2057681; rs2234767 replaced by rs7097467; rs2981578 replaced by rs10736303; rs2420946 replaced by rs2162540; rs730154 replaced by rs8031463; rs11655505 replaced by rs9646413). We further excluded rs1219648 because it was in tight LD (*R*^2^ > 0.8) with both rs2162540 and rs2981575 (Supplementary Fig. S1). Twelve SNPs that achieved genome-wide significance (*P* < 5 × 10^− 8^) for overall breast cancer in the SBCGS dataset formed the second set of SNPs (Table S2). As shown in Supplementary Fig. S2, pairwise LD analysis revealed that no pruning was needed in the second set of SNPs (*R*^2^ < 0.8). Therefore, a total of 34 SNPs were selected and genotyped in the SBCCS (Supplementary Table S1 and Supplementary Table S2).

Before genotyping, QC of DNA samples was performed and 19 samples that failed the DNA QC were excluded, resulting in a total of 807 samples (376 control participants and 431 case participants) plus 30 blind duplicate samples sent for genotyping. Genotyping of the 34 SNPs was carried out blindly by Bio Miao Biological Company Limited. Time-of-flight mass spectrometry was used for genotyping in strict accordance with a standard protocol.

QC of the SBCCS genotyping was carried out by excluding SNPs with call rate < 98%, concordance rate in duplicate samples < 99%, HWE test *P* < 0.05 (rs6730484), and SNPs that were monomorphic (Supplementary Table S1 and Supplementary Table S2). Samples were excluded if ≥3 SNPs failed the QC (6 samples were excluded). The remaining sporadic missing genotypes were imputed using population mean values.

### PRS development

The 22 SNPs in the first set of SNPs were all included from the PRS development. Of the remaining 11 SNPs in the second set, we included only two SNPs that exhibited the same effects on breast cancer in the SBCGS and SBCCS regardless of *P*-values (Supplementary Table S2). Therefore, a total of 24 SNPs were included for PRS development (Supplementary Table S3).

**Fig. 2:**
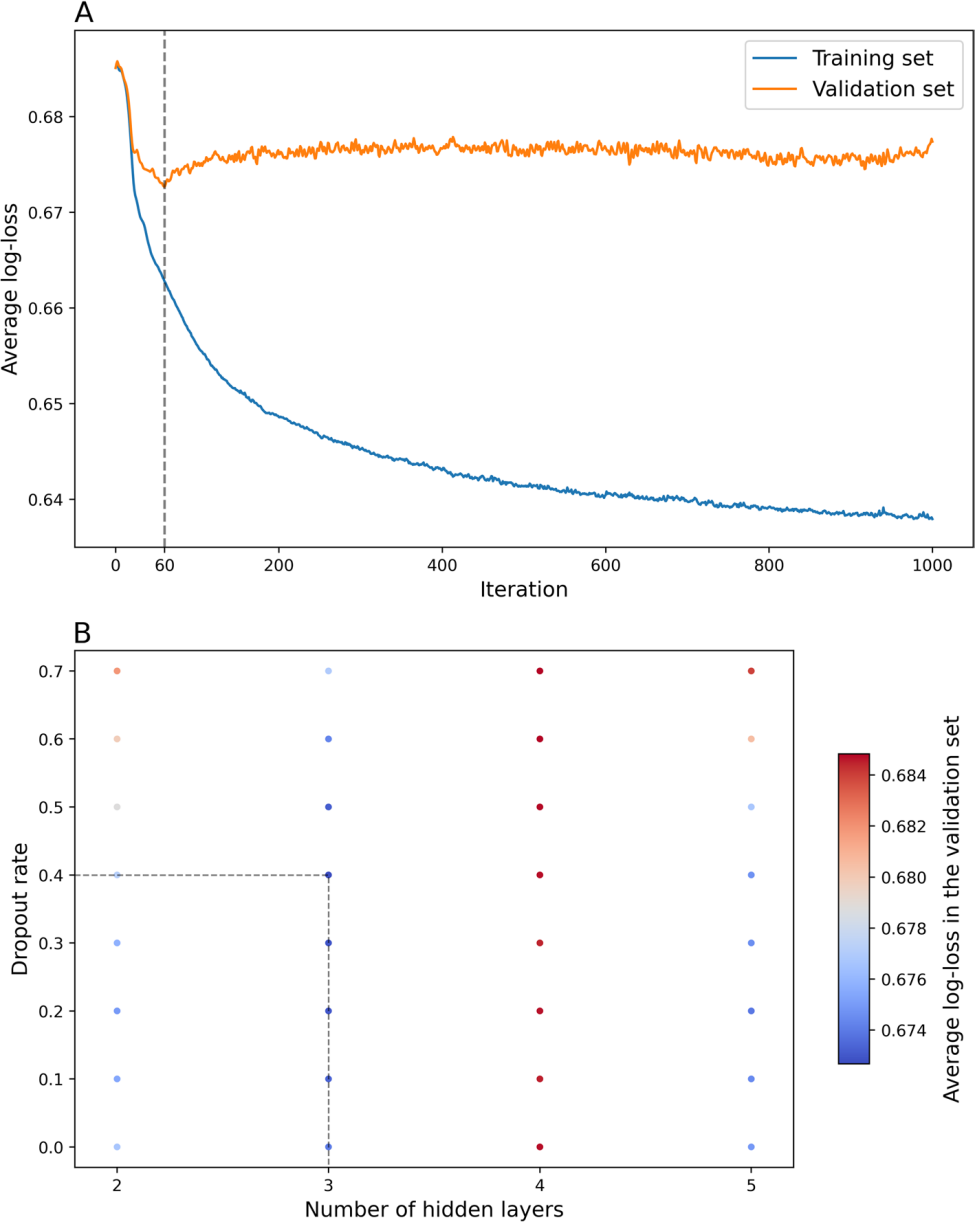
Hyperparameters tuning (**A**: number of iterations, **B**: number of hidden layers and dropout rate) results of the Artificial Neural Network model

In the current study, we used three different approaches to calculate PRSs. The first two approaches were based on the same formula: $\mathrm{PRS}=\sum_{k=1}^n{\beta}_k{x}_k$, where *n* is the total number of SNPs, *x*_*k*_ is the number of effect allele (minor allele) for the *k*th SNP, and *β*_*k*_ is the corresponding effect size, calculated as per-allele log OR for breast cancer associated with the *k*th SNP. The first approach is known as the repeated logistic regression (RLR) approach. In this approach, *β*_*k*_ was estimated in the SBCGS dataset using univariate logistic regression for each SNP individually. The RLR approach is the typical method used to calculate PRSs, since *β*_*k*_ estimated from RLR is a summary statistic and can be easily obtained without access to individual-level genotype data. In the second approach, *β*_*k*_ was estimated in the SBCGS dataset using multivariate logistic ridge regression, where all 24 SNPs were included in the model simultaneously. The model was also adjusted for age and population structure (first two principal components). The second approach is known as the logistic ridge regression (LRR) approach. The optimal penalty parameter lambda in the ridge regression model was chosen by conducting 10-fold cross-validation on the SBCGS dataset (results shown in Supplementary Fig. S3). The third approach was a newly proposed ANN-based approach. In this approach, the ANN can be considered as a perceptron, that was used to extract a vector of length 6 from the original 24 SNPs, and the final PRS was calculated based on the extracted vector while adjusting for age and population structure. The optimal hyperparameters for the ANN-based model were chosen by conducting 10-fold cross-validation on the SBCGS dataset (Fig. 2). The structure of the final ANN-based model used in the study is shown in Supplementary Fig. S4.

The primary PRSs for overall breast cancer were constructed using all breast cancer cases in the SBCGS dataset. We also constructed the PRSs for subtype-specific breast cancer (ER^+^ and ER^−^) using corresponding subtype-specific breast cancer cases in the SBCGS dataset.

Hyperparameters tuning was conducted by applying 10-fold cross validation to the SBCGS dataset and using average log-loss as the main outcome. The optimal number of iterations, hidden layers and dropout rate were 60, 3 and 0.4 respectively. Other hyperparameters that were not tuned include: number of hidden neurons in each hidden layer (square root of number of input neurons plus two); learning rate (0.01), activation function of the hidden layers (Leaky ReLU); activation function of the output layer (sigmoid); loss function (sigmoid cross entropy) and optimizer (Adam optimizer). SBCGS: Shanghai Breast Cancer Genetics Study.

### Statistical analyses

The performance of the PRSs was assessed from two perspectives: predictive ability and calibration. For predictive ability, we used the odds ratio (OR) per interquartile range (IQR) increase (IQ-OR) in the PRSs in the controls as the primary outcome. Discrimination was also used as a metric for the evaluation of predictive ability. Discrimination was assessed by the area under the receiver operator characteristic curve (AUC) with confidence intervals estimated using the Hanley and McNeil’s method [33]. To indirectly compare the predictive ability of our PRSs with previous PRSs, we also assessed the odds of breast cancer in the fourth quartile (Q_4th_) of the PRSs in controls with those in the first quartile (Q_1st_). Calibration was assessed by inspecting the observed OR to the expected OR in each PRS decile and were further estimated using coefficients from log scale linear regression as described by Brentnall et al. [23]. In addition to evaluating the crude performance of the PRSs, we also evaluated their performance after adjusting for non-genetic risk factors or absolute risks predicted by the Gail-2 model, to investigate the ability of our PRSs to provide additional risk information for Chinese women. To this end, we regressed the PRSs (as the dependent variable) against non-genetic risk factors and used the remainder of the PRSs to calculate the evaluation metrics described above. The non-genetic risk factors used for adjustment included age, age of menarche, number of live births, family history of breast cancer, body mass index (BMI), and menopausal status. Sensitivity analyses were conducted as follows: 1) by excluding samples with sporadic missing genotypes in the SBCCS dataset, and 2) by incorporating a more rigorous pruning in the SNP selection process (*R*^2^ < 0.3).

The Gail-2 model 5-year absolute risks were calculated using SAS Macro (version 4 downloaded from https://dceg.cancer.gov/tools/risk-assessment/bcrasasmacro) in SAS (version 9.4 SAS Institute Inc., Cary, NC, USA). All the statistical analyses were performed using scikit-learn (version 0.21.2), TensorFlow (version 1.13.1) and SciPy (version 1.1.0) in Python 3.6.

## Results

The age and ER status profile of the participants in SBCGS are shown in Supplementary Table S4. ER status information was available for only 1495 case participants (54.9%), among which 985 cases were ER^+^ breast cancer patients and 510 cases were ER^−^ breast cancer patients.

**Fig. 3:**
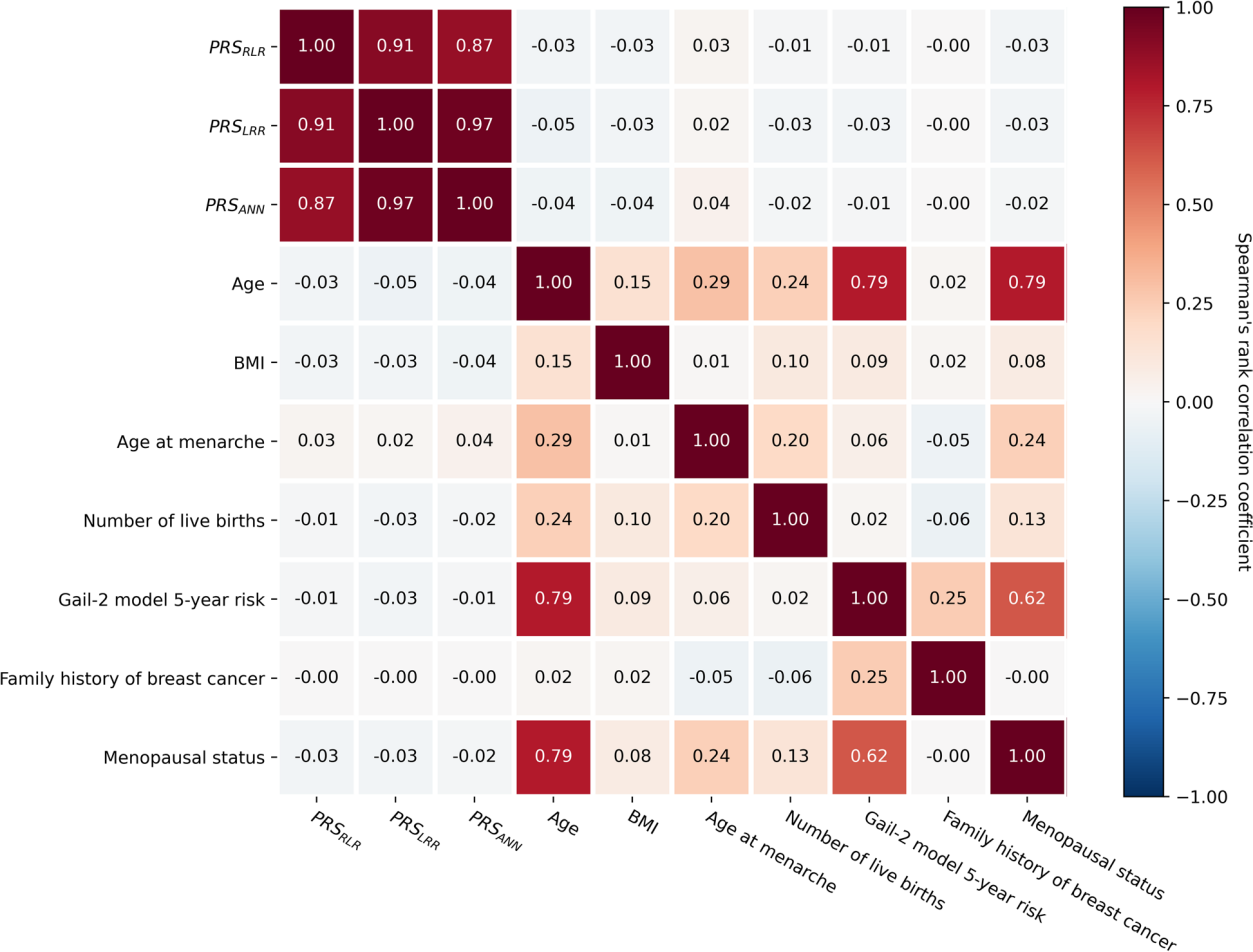
Spearman’s rank correlation coefficient matrix for breast cancer risk factors, PRSs and Gail-2 model 5-year risk. BMI: body mass index; PRS: polygenic risk score; RLR: repeated logistic regression; LRR: logistic ridge regression; ANN: Artificial Neural Network

Basic characteristics of the included 427 case and 374 control participants in the SBCCS are shown in Table 1. Due to a relatively small sample size, case and control participants were comparable in terms of several breast cancer risk factors, including BMI, age at menarche and family history of breast cancer (*P* > 0.05). Furthermore, there were no significant differences in 5-year absolute risks of breast cancer predicted by the Gail-2 model between case and control participants (*P* = 0.07). Comparison of the basic characteristics of the participants in the SBCCS who were included in the current study with those of the participants not included due to unavailability of DNA samples showed that there were no significant differences between these two groups of participants (Supplementary Table S5). As revealed by Fig. 3, the three primary PRSs for overall breast cancer (PRS_RLR_, PRS_LRR_, PRS_ANN_) had very weak correlation with other breast cancer risk factors. Associations between the PRSs and Gail-2 model 5-year risk were also very weak (Spearman’s *ρ* = − 0.01, − 0.03, and − 0.01 for PRS_RLR_, PRS_LRR_ and PRS_ANN_, respectively), suggesting that the PRSs were independent of absolute risk predicted by the Gail-2 model.

**Table 1:** Characteristic of the participants in the Sichuan Breast Cancer Case-Control Study

Characteristics	Control (***N*** = 374)	Case (***N*** = 427)	*P*-value^*^
**Continuous variables (median, IQR)**			
Age (years)	48.00 (42.00–53.00)	50.00 (44.00–57.00)	0.01
BMI (kg/m^2^)	23.37 (21.46–25.10)	22.94 (21.23–25.24)	0.18
Age at menarche (years)	14.00 (13.00–15.00)	14.00 (13.00–15.00)	0.29
Number of live births (N)	1.00 (1.00–1.00)	1.00 (1.00–2.00)	< 0.001
Gail-2 model 5-year risk (%)	0.54 (0.42–0.67)	0.54 (0.46–0.67)	0.07
PRS_RLR_	0.44 (0.05–0.84)	0.62 (0.23–1.05)	< 0.001
PRS_LRR_	0.02 (−0.18–0.23)	0.14 (− 0.06–0.32)	< 0.001
PRS_ANN_	− 0.17 (− 0.33–0.09)	0.01 (− 0.24–0.13)	< 0.001
**Categorical variables (N, %)**			
Menopausal status			0.33
*Premenopausal*	223 (59.63%)	239 (55.97%)	
*Postmenopausal*	151 (40.37%)	188 (44.03%)	
Family history of breast cancer			0.83
*Yes*	7 (1.87%)	10 (2.34%)	
*No*	367 (98.13%)	417 (97.66%)	

^*^P-value from Mann-Whitney U test (continuous variables) or chi-square test (categorical variables)

*BMI* body mass index, *IQR* interquartile range, *PRS* polygenic risk score, *RLR* repeated logistic regression, *LRR* logistic ridge regression, *ANN* Artificial Neural Network

**Fig. 4:**
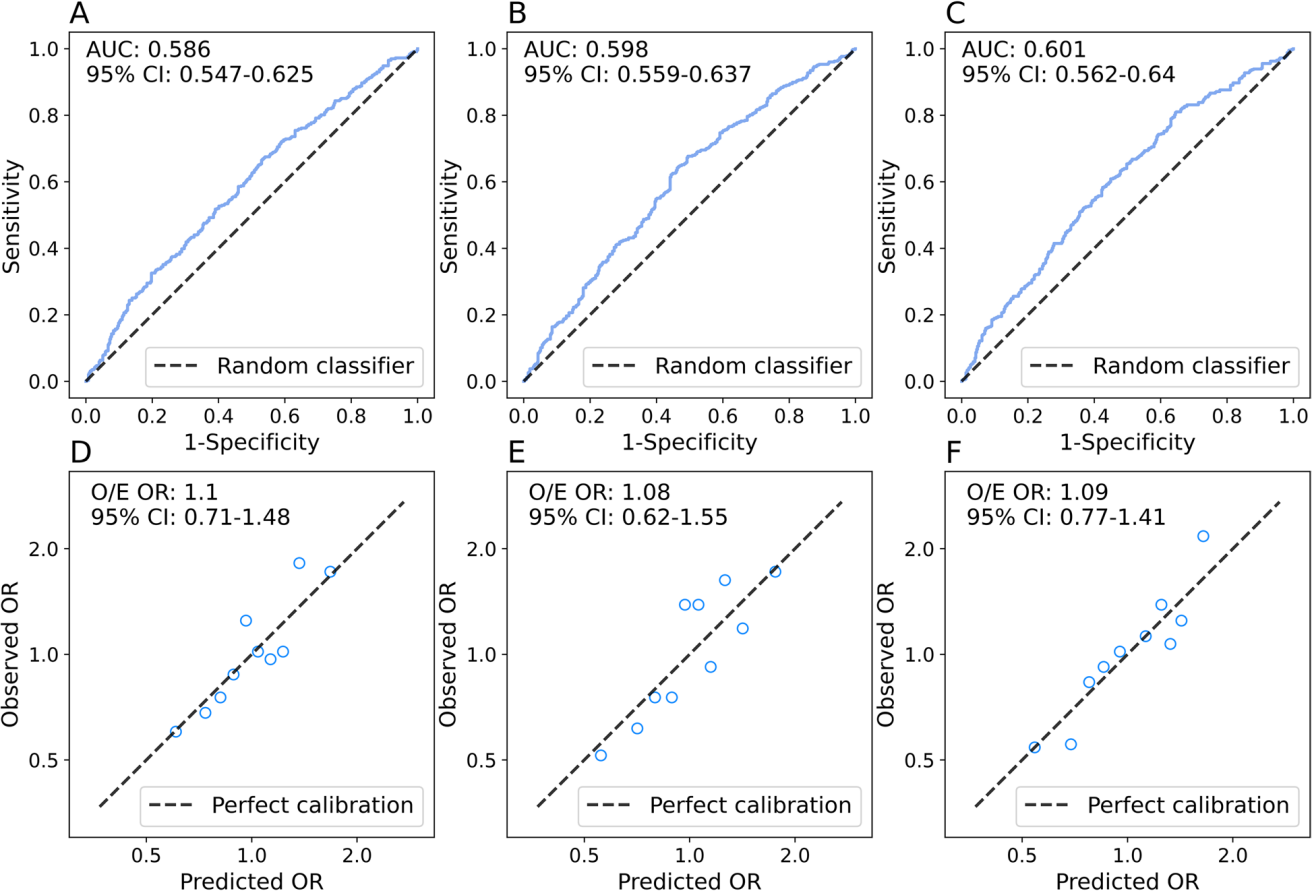
Receiver operator characteristic curves for primary unadjusted **A**: PRS_RLR_, **B**: PRS_LRR_ **C**: PRS_ANN_, and calibration plots for primary unadjusted **D**: PRS_RLR_, **E**: PRS_LRR_ **F**: PRS_ANN_. In the calibration plots, each circle represents the predicted OR and observed OR by decile. O/E OR is the ratio of the observed to expected OR, corresponding to the coefficients of the log scale linear regression. OR: odds ratio; PRS: polygenic risk score; RLR: repeated logistic regression; LRR: logistic ridge regression; ANN: Artificial Neural Network; AUC: area under the receiver operator characteristic curve

For overall breast cancer, the primary PRSs constructed using the ANN-based approach achieved higher IQ-OR (1.76, 95% CI 1.39–2.24) than the primary PRSs constructed using RLR (IQ-OR 1.49, 95% CI 1.23–1.81) and LRR (IQ-OR 1.58, 95% CI 1.29–1.92, Table 2). In terms of discrimination (Fig. 4), PRS_LRR_ and PRS_ANN_ were comparable (AUC 0.598, 95% CI 0.559–0.637 vs. AUC 0.601, 95% CI 0.562–0.640) and superior to PRS_RLR_ (AUC 0.582, 95% CI 0.543–0.621). As shown in Fig. 4, all three PRSs were well calibrated to overall breast cancer relative risks in Chinese women, with the observed to expected OR (O/E OR) of 1.10 (95% CI 0.71–1.48), 1.08 (95% CI 0.62–1.55) and 1.09 (95% CI 0.77–1.41) for PRS_RLR_, PRS_LRR_ and PRS_ANN_, respectively. The primary PRSs showed slightly better predictive ability for ER^+^ breast cancer but significantly poorer predictive ability for ER^−^ breast cancer. For ER^+^ breast cancer, PRS_ANN_ (IQ-OR 1.96, 95% CI 1.50–2.55; AUC 0.620, 95% CI 0.577–0.663) outperformed PRS_RLR_ and PRS_LRR_ in terms of predictive ability. Calibration of the PRSs for ER^+^ breast cancer remained similar. For ER^−^ breast cancer, the primary PRSs had similarly poor IQ-OR (1.27–1.32) and AUC (0.550–0.555). PRS_ANN_ was poorly calibrated to ER- breast cancer risks (O/E OR 1.37, 95% CI -0.62–3.35). Adjustment for the Gail-2 model absolute risks had almost no effect on the performance of the primary PRSs (results shown in Supplementary Table S6), while adjustment for the breast cancer risk factors slightly reduced the predictive ability of the PRSs (Table 2).

**Table 2:** Predictive performance of the primary PRSs for overall and ER^+^/ER^−^ breast cancer

PRS	N (Controls/Cases)	Cases (Median, IQR)	Controls (Median, IQR)	Q_**4th**_ vs Q_**1st**_ OR (95% CI)	IQ-OR (95% CI)	O/E OR (95% CI)	AUC (95% CI)	Adjustment^a^
**Overall breast cancer**								
PRS_RLR_	374/427	0.62 (0.23–1.05)	0.44 (0.05–0.84)	2.09 (1.40–3.11)	1.49 (1.23–1.81)	1.10 (0.71–1.48)	0.586 (0.547–0.625)	None
		0.15 (−0.23–0.58)	−0.01 (− 0.40–0.36)	2.02 (1.36–2.99)	1.43 (1.19–1.72)	1.08 (0.51–1.64)	0.582 (0.543–0.621)	B
PRS_LRR_		0.14 (−0.06–0.32)	0.02 (− 0.18–0.23)	2.59 (1.71–3.91)	1.58 (1.29–1.92)	1.08 (0.62–1.55)	0.598 (0.559–0.637)	None
		0.11 (−0.09–0.28)	− 0.01 (− 0.22–0.21)	2.47 (1.63–3.73)	1.57 (1.28–1.93)	1.08 (0.81–1.35)	0.595 (0.556–0.634)	B
PRS_ANN_		0.01 (− 0.24–0.13)	−0.17 (− 0.33–0.09)	2.61 (1.72–3.95)	1.76 (1.39–2.24)	1.09 (0.77–1.41)	0.601 (0.562–0.640)	None
		0.15 (−0.10–0.26)	−0.02 (− 0.19–0.22)	2.51 (1.67–3.79)	1.68 (1.34–2.12)	1.17 (0.62–1.72)	0.596 (0.557–0.635)	B
**ER**^b^ **breast cancer**								
PRS_RLR_	374/290	0.65 (0.24–1.06)	0.44 (0.05–0.84)	2.24 (1.44–3.48)	1.56 (1.26–1.93)	1.09 (0.61–1.57)	0.597 (0.553–0.641)	None
		0.20 (−0.22–0.60)	− 0.01 (− 0.40–0.36)	2.18 (1.41–3.37)	1.49 (1.22–1.83)	1.06 (0.53–1.59)	0.592 (0.548–0.636)	B
PRS_LRR_		0.15 (− 0.05–0.33)	0.02 (− 0.18–0.23)	2.94 (1.85–4.69)	1.67 (1.34–2.08)	1.12 (0.74–1.51)	0.613 (0.570–0.656)	None
		0.12 (− 0.07–0.30)	−0.01 (− 0.22–0.21)	2.72 (1.71–4.32)	1.67 (1.33–2.10)	1.10 (0.75–1.45)	0.608 (0.565–0.651)	B
PRS_ANN_		0.04 (−0.22–0.15)	− 0.17 (− 0.33–0.09)	3.00 (1.87–4.78)	1.96 (1.50–2.55)	1.09 (0.80–1.38)	0.620 (0.577–0.663)	None
		0.17 (− 0.09–0.28)	−0.02 (− 0.19–0.22)	2.89 (1.82–4.58)	1.85 (1.43–2.39)	1.12 (0.65–1.59)	0.614 (0.571–0.657)	B
**ER**^**−**^ **breast cancer**								
PRS_RLR_	374/124	0.57 (0.19–0.92)	0.44 (0.05–0.84)	1.63 (0.91–2.95)	1.27 (0.96–1.69)	1.08 (0.42–1.74)	0.554 (0.495–0.613)	None
		0.10 (−0.27–0.49)	− 0.01 (− 0.40–0.36)	1.53 (0.86–2.73)	1.24 (0.94–1.62)	1.17 (0.35–1.99)	0.549 (0.490–0.608)	B
PRS_LRR_		0.08 (− 0.11–0.28)	0.02 (− 0.18–0.23)	1.79 (0.98–3.28)	1.29 (0.97–1.72)	1.23 (0.18–2.28)	0.555 (0.496–0.614)	None
		0.05 (− 0.12–0.25)	− 0.01 (− 0.22–0.21)	1.87 (1.00–3.50)	1.30 (0.96–1.75)	1.15 (0.41–1.89)	0.555 (0.496–0.614)	B
PRS_ANN_		−0.13 (− 0.25–0.11)	−0.17 (− 0.33–0.09)	1.78 (0.96–3.30)	1.32 (0.93–1.87)	1.37 (− 0.62–3.35)	0.550 (0.491–0.609)	None
		−0.01 (− 0.11–0.24)	−0.02 (− 0.19–0.22)	1.70 (0.92–3.14)	1.30 (0.93–1.82)	1.21 (− 0.18–2.60)	0.548 (0.489–0.607)	B

^a^Adjustment B: adjusted for classical breast cancer risk factors; Q_4th_ vs Q_1st_ OR: participants in the fourth quartile of the PRS vs those in the first quartile

*ER*^b^ estrogen receptor positive, *ER* estrogen receptor negative, *OR* odds ratio, *Q*_*4th*_ fourth quartile, *Q*_*1st*_ first quartile, *IQR* interquartile range, *PRS* polygenic risk score, *RLR* repeated logistic regression, *LRR* logistic ridge regression, *ANN* Artificial Neural Network, *IQ-OR* OR per IQR increase of the PRS in controls, *O/E OR* observed to expected OR, *AUC* area under the receiver operator characteristic curve

The performance of the subtype-specific PRSs is shown in Table 3. In general, ER^+^ PRS_RLR_ and ER^+^ PRS_LRR_ showed similar performance to the corresponding primary PRSs of ER^+^ breast cancer, whereas the performance of the ER^+^ PRS_ANN_ of ER^+^ breast cancer was worse than that of the primary PRS_ANN_ (IQ-OR 1.60 vs. 1.96; AUC 0.612 vs. 0.620; O/E OR 1.16 vs. 1.09). Compared with the primary PRSs, all ER^−^ PRSs showed substantial improvement in the prediction of ER^−^ breast cancer. Among the three ER^−^ PRSs, ER^−^ PRS_LRR_ achieved the highest predictive ability (IQ-OR 1.52 95% CI 1.10–2.10; AUC 0.582 95% CI 0.523–0.641) but still underestimated the ER^−^ breast cancer risk to some extent (O/E OR 1.13 95% CI 0.04–2.21). Adjustment for the Gail-2 model absolute risks and breast cancer risk factors had limited effect on the performance of ER^+^/ER^−^ PRS_RLR_ and PRS_LRR_, which was similar to that observed in the primary PRSs. However, adjustment for breast cancer risk factors substantially reduced the predictive and discriminative abilities of the ER^+^/ER^−^ PRS_ANN_.

**Table 3:** Predictive performance of the subtype-specific PRSs for ER^+^/ER^−^ breast cancer

PRS	N (Controls/Cases)	Cases (Median, IQR)	Controls (Median, IQR)	Q_**4th**_ vs Q_**1st**_ OR (95% CI)	IQ-OR (95% CI)	O/E OR (95% CI)	AUC (95% CI)	Adjustment^a^
**ER**^b^ **breast cancer**								
ER^b^ PRS_RLR_	374/290	0.65 (0.19–1.08)	0.41 (0.02–0.85)	2.23 (1.43–3.46)	1.55 (1.25–1.91)	1.05 (0.73–1.37)	0.596 (0.552–0.640)	None
		0.22 (−0.24–0.65)	− 0.02 (− 0.42–0.42)	2.22 (1.43–3.46)	1.55 (1.25–1.92)	1.07 (0.75–1.39)	0.595 (0.551–0.639)	A
		0.20 (− 0.26–0.62)	−0.02 (− 0.40–0.40)	1.94 (1.26–2.99)	1.48 (1.21–1.81)	1.05 (0.53–1.56)	0.591 (0.547–0.635)	B
ER^b^ PRS_LRR_		0.11 (−0.09–0.29)	−0.03 (− 0.23–0.18)	2.92 (1.86–4.59)	1.70 (1.37–2.10)	1.09 (0.76–1.42)	0.618 (0.575–0.661)	None
		0.12 (−0.06–0.31)	−0.01 (− 0.21–0.20)	2.93 (1.86–4.63)	1.69 (1.36–2.09)	1.07 (0.69–1.46)	0.617 (0.574–0.660)	A
		0.13 (−0.08–0.31)	−0.01 (− 0.20–0.21)	2.49 (1.59–3.89)	1.65 (1.33–2.04)	1.06 (0.71–1.41)	0.613 (0.570–0.656)	B
ER^b^ PRS_ANN_		−0.11 (− 0.26–0.00)	−0.21 (− 0.40–0.07)	2.81 (1.79–4.42)	1.60 (1.28–2.01)	1.16 (0.56–1.75)	0.612 (0.569–0.655)	None
		0.14 (−0.01–0.26)	0.05 (− 0.13–0.19)	2.51 (1.62–3.90)	1.57 (1.26–1.95)	1.18 (0.53–1.84)	0.611 (0.568–0.654)	A
		0.13 (−0.02–0.24)	0.05 (− 0.15–0.18)	2.44 (1.57–3.81)	1.47 (1.18–1.83)	1.19 (0.72–1.66)	0.596 (0.552–0.640)	B
**ER**^**−**^ **breast cancer**								
ER^−^ PRS_RLR_	374/124	0.68 (0.31–0.97)	0.52 (0.15–0.83)	1.84 (1.02–3.33)	1.41 (1.04–1.90)	1.26 (0.52–2.01)	0.574 (0.515–0.633)	None
		0.15 (−0.22–0.44)	0.00 (− 0.37–0.31)	1.91 (1.05–3.48)	1.41 (1.04–1.90)	1.28 (0.42–2.15)	0.573 (0.514–0.632)	A
		0.12 (−0.23–0.45)	0.01 (− 0.35–0.33)	1.68 (0.94–3.02)	1.38 (1.02–1.86)	1.29 (0.43–2.15)	0.570 (0.511–0.629)	B
ER^−^ PRS_LRR_		0.23 (0.10–0.44)	0.16 (−0.05–0.37)	2.55 (1.34–4.88)	1.52 (1.10–2.10)	1.13 (0.04–2.21)	0.582 (0.523–0.641)	None
		0.08 (−0.06–0.29)	0.00 (− 0.21–0.21)	2.55 (1.31–4.93)	1.52 (1.10–2.09)	1.21 (0.08–2.33)	0.582 (0.523–0.641)	A
		0.07 (−0.07–0.28)	0.00 (− 0.20–0.20)	2.26 (1.18–4.33)	1.47 (1.08–1.99)	1.13 (0.26–2.00)	0.578 (0.519–0.637)	B
ER^−^ PRS_ANN_		−0.12 (− 0.25–0.02)	−0.16 (− 0.37–0.03)	2.10 (1.09–4.07)	1.52 (1.09–2.12)	1.29 (0.74–1.84)	0.562 (0.503–0.621)	None
		0.10 (−0.02–0.21)	0.06 (− 0.14–0.20)	2.22 (1.15–4.30)	1.52 (1.09–2.12)	1.28 (0.61–1.95)	0.562 (0.503–0.621)	A
		0.09 (−0.05–0.2)	0.06 (− 0.15–0.20)	1.90 (0.99–3.62)	1.38 (0.99–1.93)	1.16 (0.34–1.98)	0.547 (0.488–0.606)	B

^a^Adjustment A: adjusted for Gail-2 model 5-year absolute risk, adjustment B: adjusted for classical breast cancer risk factors

*ER*^b^ estrogen receptor positive, *ER* estrogen receptor negative, *OR* odds ratio, *Q*_*4th*_ fourth quartile, *Q*_*1st*_ first quartile, *IQR* interquartile range, *PRS* polygenic risk score, *RLR* repeated logistic regression, *LRR* logistic ridge regression, *ANN* Artificial Neural Network, *IQ-OR* OR per IQR increase of the PRS in controls, *O/E OR* observed to expected OR, *AUC* area under the receiver operator characteristic curve

The sensitivity analysis conducted by excluding samples with missing genotypes in the SBCCS dataset did not reveal significant changes in the main results (Supplementary Table S7). The ANN-based and LRR approaches can compensate for the issue of collinearity; therefore, we incorporated a loose *R*^2^ threshold of 0.8 when selecting the SNPs in order to include more informative SNPs. However, this threshold may have influenced the performance of the PRS_RLR_. A sensitivity analysis was conducted by incorporating a more rigorous *R*^2^ threshold, which led to the removal of seven additional SNPs (rs2981582, rs3803662, rs9646413, rs2162540, rs10736303, rs4479849, and rs10789190). The performance of the PRS_RLR_ constructed using SNP-17 was slightly improved but did not exceed the performance of the primary PRS_LRR_ and PRS_ANN_ (Supplementary Table S8).

## Discussion

In the current study, the PRSs for the prediction of overall breast cancer and subtype-specific breast cancer in Chinese women were developed using a GWAS dataset and validated in an external case-control dataset. The best PRSs (PRS_ANN_ and PRS_LRR_) based on 24 SNPs showed modest predictive ability (PRS_ANN_: IQ-OR 1.76; AUC 0.601; PRS_LRR_: IQ-OR 1.58; AUC 0.598) and calibration (PRS_ANN_: O/E OR 1.09; PRS_LRR_: O/E OR 1.08) for overall breast cancer. More importantly, the study results showed that the PRS_ANN_ was largely independent of Gail-2 model 5-year risk and other non-genetic risk factors. The PRS_ANN_ remained predictive of overall breast cancer after adjustment for classical breast cancer risk factors (PRS_ANN_: IQ-OR 1.68; AUC 0.596; PRS_LRR_: IQ-OR 1.57; AUC 0.595), although the calibration index seemed to be slightly altered for PRS_ANN_ (O/E OR 1.17). These results indicated that our PRSs can provide additional risk information and are therefore suitable for use in conjunction with breast cancer risk prediction models based on non-genetic risk factors to stratify women into different risk groups. Since the Gail-2 model is the only publicly available model that can be implemented in our dataset, we also investigated the combination of the PRS_ANN_ and PRS_LRR_ with the Gail-2 model. Although there was a substantial increase in AUC when the PRSs were combined with the Gail-2 model (increased from 0.531 to approximately 0.58), the combined models had lower predictive ability than that using the PRSs alone. This was largely due to the poor performance of the Gail-2 model in the SBCCS dataset, which was consistent with a recent meta-analysis reporting a pooled AUC of 0.55 (95% CI 0.52–0.58) for the Gail-2 model in Asian females [34]. Therefore, although our PRSs showed great potential to contribute additional risk information and increase predictive ability when combined with classical breast cancer risk factors, further studies are still needed to investigate their performance when combined with a more accurate non-genetic risk prediction model for Chinese women (e.g., Han Chinese Breast Cancer Prediction model [35]).

Another important application of the PRS is to identify women at high risk of breast cancer who could benefit from more frequent breast cancer screening or preventive therapy. Therefore, it is also important to assess the ability of the PRSs in predicting risk in the tails of the distribution. In the current study, the adjusted Q_4th_ vs. Q_1st_ ORs for PRS_ANN_ and PRS_LRR_ were 2.51 and 2.47, respectively, meaning women in the fourth quartiles of the PRSs had an approximately 2.5-fold greater risk of having breast cancer than those in the lowest quartiles. This represents a substantial improvement in predictive ability compared with previous PRSs developed for Chinese women (Supplementary Table S9). This improvement can be attributed to the use of individual-level genotype data and a more sophisticated approach for PRS construction. Nevertheless, our best PRSs were still less predictive compared with some recent PRSs developed for women of European ancestry [22, 23, 36, 37], perhaps reflecting the gap between the number of SNPs included. Therefore, the performance of these PRSs can still be improved by including more SNPs associated with breast cancer risk in the Chinese population.

Previous studies conducted in women of European ancestry showed that breast cancer PRSs were generally less predictive of ER^−^ breast cancer than ER^+^ breast cancer [22]. We confirmed this result in our dataset in the Chinese population. The primary PRSs were significantly less predictive and poorly calibrated for ER^−^ breast cancer, with adjusted IQ-OR and AUCs ranging from 1.24 to 1.30 and 0.548 to 0.555, respectively. To improve the prediction of ER^−^ breast cancer, we developed ER^−^ PRSs in the current study. The results indicated that when training PRSs solely on ER^−^ breast cancer cases yielded a substantial gain in predictive ability for ER^−^ breast cancer. As a more aggressive breast cancer subtype, patients with ER^−^ breast cancer had significantly worse prognosis compared with patients with ER^+^ breast cancer. Identifying women at high risk of ER^−^ breast cancer regardless of their overall breast cancer risk is therefore of great value in clinical practice and breast cancer screening. Our results highlighted the requirement for optimization of future PRS for ER^−^ breast cancer by incorporating more ER^−^ cases in the training dataset and perhaps, including more SNPs associated with ER^−^ breast cancer.

We compared three different approaches to PRS construction, consisting of the traditional RLR approach using summary statistics, as well as LRR approach and the newly proposed ANN-based approach using individual-level genotype data. Compared with the traditional summary statistics-based RLR approach, the LRR and ANN approaches can be used to address the issues of overfitting, collinearity and confounding by using individual-level genotype data, thus providing a more accurate estimate of the weighting parameters. Therefore, it is expected that the primary PRSs constructed using the ANN and LRR approaches both achieved better predictive performance than PRS_RLR_ (including SNP-17 based PRS_RLR_ in the sensitivity analysis). Through the use of the non-linear activation function and multiple hidden layers, the ANN model is able to fit high-order interactions between variables [38]. Therefore, in theory, the ANN approach captures the interactions between breast cancer SNPs [39–41], and thereby achieves better predictive performance than the linear LRR approach. Our research confirms this speculation. The primary PRS_ANN_ showed better predictive ability than the primary PRS_LRR_ in predicting overall and ER^+^ breast cancer, which suggests the existence of interactions between the included SNPs. To explore possible SNP-SNP interactions, we conducted logistic regression analyses to identify pairwise interactions in the SBCGS dataset. A total of 13 pairs of SNPs with possible SNP-SNP interactions (*P* < 0.05) were identified, but none of them reached a Bonferroni corrected level of statistical significance (*P* < 1.8 × 10^− 4^). Further *post-hoc* analysis revealed that the interaction between rs10789190 and rs7799039 was statistically significant in both datasets (*P* < 0.05). The SNPs rs10789190 and rs7799039 are located in the leptin (LEP) and leptin receptor (LEPR) genes, respectively, making their interaction biologically plausible. Adding this interaction term to the PRS_LRR_ slightly improved its predictive ability (PRS_LRR_ with interaction term: IQ-OR 1.62; AUC 0.602), indicating the differences between the primary PRS_LRR_ and PRS_LRR_ can be partially attributed to this interaction term. In other words, the ANN approach automatically captures the potential interactions between SNPs, which are likely to be omitted in the traditional approaches. Nevertheless, the ANN approach is more sophisticated and less flexible than the LRR and RLR approaches. Whether ANN can be considered the optimal approach to PRS construction remains to be investigated.

The current study has several strengths. First, the PRSs were validated in an external dataset, and thus avoided the concern of overfitting. Nevertheless, further validation with an expanded sample, preferably from multiple locations in China, is still needed. Second, we examined the performance of the PRSs by ER status and further optimized the PRSs for ER^−^ breast cancer prediction, which has not been previously conducted in Chinese women. Future studies may consider both ER and human epidermal growth factor receptor 2 (HER2) status when optimizing the PRS for prediction of subtype breast cancer. Third, all the SNPs in the SBCGS and SBCCS were genotyped directly. Imputation was conducted only for sporadic missing genotypes. However, the study also has some limitations. First, the overall performance of the PRSs is not ideal, especially compared with the performance of PRS in women of European ancestry. Due to budget constraints, the search for candidate SNPs was limited to those that are well-validated in Chinese population, hence some newly identified SNPs and SNPs that remain to be validated in Chinese population were omitted. Therefore, the results of our study should be interpreted with caution. Future studies should include more SNPs associated with breast cancer susceptibility, especially those identified in recent GWASs. High-quality genetic studies are also needed to identify and validate more breast cancer susceptibility SNPs in the Chinese population. Second, assessment of the performance of the PRSs in combination with classical breast cancer risk factors was not sufficient, since there is no suitable breast cancer risk prediction model for Chinese women. Further studies are warranted to investigate the performance of the PRSs when incorporated into more accurate risk prediction models for Chinese women. Third, BRCA status information is unavailable for either SBCGS or SBCCS, we are therefore not able to conduct further stratified analyses or make comparisons. Besides, since the SBCGS spanned a long period of time (i.e., 1996 to 2015), we cannot rule out the possibility that changes in recommendations for determining ER status may have influenced the results of our study. Finally, our PRSs and study results are limited to Han Chinese women and may not be generalizable to Chinese women in other ethnic groups, although they only account for around 9% of the total population.

In summary, the SNP-24-based breast cancer PRSs showed significantly better predictive ability than previous PRSs developed for Chinese women. Our SNP-24-based PRSs were largely independent of classical breast cancer risk factors and thus have great potential to improve clinical practice and future risk-based breast cancer screening programs by providing additional risk information for the general population. Nevertheless, the predictive performance of the current PRSs is not ideal and can be improved by incorporating more SNPs that are associated with breast cancer risk in Chinese women. Although the subtype-specific PRSs showed substantial improvement for ER^−^ breast cancer prediction, the overall performance is still poor and improvements are still needed before it can be implemented to identify women at high risk of ER^−^ breast cancer. Our newly proposed ANN-based PRS construction approach automatically captures the potential interactions between SNPs and showed better performance than the traditional approaches, although additional studies are needed to further validate and investigate this approach.

## Supplementary Information

**Additional file 1: Table S1.** Basic information of the first set of SNPs along with the SNP selection results. **Table S2.** Basic information of the second set of SNPs along with SNP selection results. **Table S3.** Basic information of the 24 SNPs included for PRSs construction. **Table S4.** Characteristics of the participants in the SBCGS. **Table S5** Comparison between the participants in the SBCCS who were included in the current study with the participants excluded due to unavailability of DNA samples or number of failed SNPs ≥3. **Table S6.** Predictive performance of the primary PRSs for overall and ER+/ER- breast cancer. **Table S7.** Sensitivity analysis 1 - predictive performance of the primary PRSs for overall and ER+/ER- breast cancer when samples with missing genotypes were all excluded. **Table S8** Sensitivity analysis 2 - predictive performance of the SNP-17 PRS_RLR_ for overall and ER+/ER- breast cancer. **Table S9.** Indirect comparison with previous PRSs for Chinese women. **Fig. S1.** Linkage disequilibrium matrix of the first set of SNPs. **Fig. S2.** Linkage disequilibrium matrix of the second set of SNPs. **Fig. S3.** Hyperparameters tuning results of the logistic ridge regression model. **Fig. S4.** The structure of the ANN model used in the current study.

## Data Availability

The SBCGS dataset (phs000799.v1.p1) is not publicly available according to the rules and regulations of dbGaP (https://www.ncbi.nlm.nih.gov/projects/gap/cgi-bin/about.cgi), but can be applied for access; the SBCCS dataset is available from the corresponding author (JYL) on reasonable request.

## References

[1] Siegel RL, Miller KD, Jemal A (2020). Cancer statistics, 2020. CA Cancer J Clin.

[2] GBD 2017 DALYs and HALE Collaborators (2018). Global, regional, and national disability-adjusted life-years (DALYs) for 359 diseases and injuries and healthy life expectancy (HALE) for 195 countries and territories, 1990-2017: a systematic analysis for the global burden of disease study 2017. Lancet.

[3] Fan L, Strasser-Weippl K, Li JJ, St Louis J, Finkelstein DM, Yu KD, Chen WQ, Shao ZM, Goss PE (2014). Breast cancer in China. Lancet Oncol.

[4] Collaborative Group on Hormonal Factors in Breast Cancer (2012). Menarche, menopause, and breast cancer risk: individual participant meta-analysis, including 118 964 women with breast cancer from 117 epidemiological studies. Lancet Oncol.

[5] Hamajima N, Hirose K, Tajima K, Rohan T, Calle EE, Heath CW, Coates RJ, Liff JM, Talamini R, Chantarakul N (2002). Alcohol, tobacco and breast cancer--collaborative reanalysis of individual data from 53 epidemiological studies, including 58,515 women with breast cancer and 95,067 women without the disease. Br J Cancer.

[6] Yuan X, Yi F, Hou C, Lee H, Zhong X, Tao P, Li H, Xu Z, Li J (2019). Induced abortion, birth control methods, and breast Cancer risk: a case-control study in China. J Epidemiol.

[7] Chan DSM, Abar L, Cariolou M, Nanu N, Greenwood DC, Bandera EV, McTiernan A, Norat T (2019). World Cancer research fund international: continuous update project-systematic literature review and meta-analysis of observational cohort studies on physical activity, sedentary behavior, adiposity, and weight change and breast cancer risk. Cancer Causes Control.

[8] Ho PJ, Lau HSH, Ho WK, Wong FY, Yang Q, Tan KW, Tan MH, Chay WY, Chia KS, Hartman M (2020). Incidence of breast cancer attributable to breast density, modifiable and non-modifiable breast cancer risk factors in Singapore. Sci Rep.

[9] Mavaddat N, Antoniou AC, Easton DF, Garcia-Closas M (2010). Genetic susceptibility to breast cancer. Mol Oncol.

[10] Walsh T, Casadei S, Coats KH, Swisher E, Stray SM, Higgins J, Roach KC, Mandell J, Lee MK, Ciernikova S (2006). Spectrum of mutations in BRCA1, BRCA2, CHEK2, and TP53 in families at high risk of breast cancer. JAMA.

[11] Peto J, Collins N, Barfoot R, Seal S, Warren W, Rahman N, Easton DF, Evans C, Deacon J, Stratton MR (1999). Prevalence of BRCA1 and BRCA2 gene mutations in patients with early-onset breast cancer. J Natl Cancer Inst.

[12] Suter NM, Ray RM, Hu YW, Lin MG, Porter P, Gao DL, Zaucha RE, Iwasaki LM, Sabacan LP, Langlois MC (2004). BRCA1 and BRCA2 mutations in women from Shanghai China. Cancer Epidemiol Biomarkers Prev.

[13] Yanes T, Young MA, Meiser B, James PA (2020). Clinical applications of polygenic breast cancer risk: a critical review and perspectives of an emerging field. Breast Cancer Res.

[14] Pashayan N, Duffy SW, Chowdhury S, Dent T, Burton H, Neal DE, Easton DF, Eeles R, Pharoah P (2011). Polygenic susceptibility to prostate and breast cancer: implications for personalised screening. Br J Cancer.

[15] Hughes E, Judkins T, Wagner S, Wenstrup R, Lanchbury JS, Gutlin A (2017). Development and validation of a residual risk score to predict breast cancer risk in unaffected women negative for mutations on a multi-gene hereditary cancer panel. J Clin Oncol.

[16] Pashayan N, Morris S, Gilbert FJ, Pharoah PDP (2018). Cost-effectiveness and benefit-to-harm ratio of risk-stratified screening for breast Cancer: a life-table model. JAMA Oncol.

[17] Zheng W, Wen W, Gao YT, Shyr Y, Zheng Y, Long J, Li G, Li C, Gu K, Cai Q (2010). Genetic and clinical predictors for breast cancer risk assessment and stratification among Chinese women. J Natl Cancer Inst.

[18] Dai J, Hu Z, Jiang Y, Shen H, Dong J, Ma H, Shen H (2012). Breast cancer risk assessment with five independent genetic variants and two risk factors in Chinese women. Breast Cancer Res.

[19] Lee CP, Irwanto A, Salim A, Yuan JM, Liu J, Koh WP, Hartman M (2014). Breast cancer risk assessment using genetic variants and risk factors in a Singapore Chinese population. Breast Cancer Res.

[20] Hsieh YC, Tu SH, Su CT, Cho EC, Wu CH, Hsieh MC, Lin SY, Liu YR, Hung CS, Chiou HY (2017). A polygenic risk score for breast cancer risk in a Taiwanese population. Breast Cancer Res Treat.

[21] Chan CHT, Munusamy P, Loke SY, Koh GL, Yang AZY, Law HY, Yoon CS, Wong CY, Yong WS, Wong NS (2018). Evaluation of three polygenic risk score models for the prediction of breast cancer risk in Singapore Chinese. Oncotarget.

[22] Mavaddat N, Michailidou K, Dennis J, Lush M, Fachal L, Lee A, Tyrer JP, Chen TH, Wang Q, Bolla MK (2019). Polygenic risk scores for prediction of breast Cancer and breast Cancer subtypes. Am J Hum Genet.

[23] Brentnall AR, van Veen EM, Harkness EF, Rafiq S, Byers H, Astley SM, Sampson S, Howell A, Newman WG, Cuzick J (2020). A case-control evaluation of 143 single nucleotide polymorphisms for breast cancer risk stratification with classical factors and mammographic density. Int J Cancer.

[24] Wen W, Shu XO, Guo X, Cai Q, Long J, Bolla MK, Michailidou K, Dennis J, Wang Q, Gao YT (2016). Prediction of breast cancer risk based on common genetic variants in women of east Asian ancestry. Breast Cancer Res.

[25] Gao YT, Shu XO, Dai Q, Potter JD, Brinton LA, Wen W, Sellers TA, Kushi LH, Ruan Z, Bostick RM (2000). Association of menstrual and reproductive factors with breast cancer risk: results from the Shanghai breast Cancer study. Int J Cancer.

[26] Dorjgochoo T, Gu K, Kallianpur A, Zheng Y, Zheng W, Chen Z, Lu W, Shu XO (2009). Menopausal symptoms among breast cancer patients 6 months after diagnosis: a report from the Shanghai breast Cancer survival study. Menopause.

[27] Matthews CE, Xu WH, Zheng W, Gao YT, Ruan ZX, Cheng JR, Xiang YB, Shu XO (2005). Physical activity and risk of endometrial cancer: a report from the Shanghai endometrial cancer study. Cancer Epidemiol Biomarkers Prev.

[28] Zheng W, Chow WH, Yang G, Jin F, Rothman N, Blair A, Li HL, Wen W, Ji BT, Li Q (2005). The Shanghai Women's health study: rationale, study design, and baseline characteristics. Am J Epidemiol.

[29] Manichaikul A, Mychaleckyj JC, Rich SS, Daly K, Sale M, Chen WM (2010). Robust relationship inference in genome-wide association studies. Bioinformatics.

[30] Howie B, Fuchsberger C, Stephens M, Marchini J, Abecasis GR (2012). Fast and accurate genotype imputation in genome-wide association studies through pre-phasing. Nat Genet.

[31] Chang CC, Chow CC, Tellier LC, Vattikuti S, Purcell SM, Lee JJ (2015). Second-generation PLINK: rising to the challenge of larger and richer datasets. GigaScience.

[32] Machiela MJ, Chanock SJ (2015). LDlink: a web-based application for exploring population-specific haplotype structure and linking correlated alleles of possible functional variants. Bioinformatics.

[33] Hanley JA, McNeil BJ (1982). The meaning and use of the area under a receiver operating characteristic (ROC) curve. Radiology.

[34] Wang X, Huang Y, Li L, Dai H, Song F, Chen K (2018). Assessment of performance of the Gail model for predicting breast cancer risk: a systematic review and meta-analysis with trial sequential analysis. Breast Cancer Res.

[35] Wang L, Liu L, Lou Z, Ding L, Guan H, Wang F, Yu L, Xiang Y, Zhou F, Xue F (2019). Risk prediction for breast Cancer in Han Chinese women based on a cause-specific Hazard model. BMC Cancer.

[36] Cuzick J, Brentnall AR, Segal C, Byers H, Reuter C, Detre S, Lopez-Knowles E, Sestak I, Howell A, Powles TJ (2017). Impact of a panel of 88 single nucleotide polymorphisms on the risk of breast Cancer in high-risk women: results from two randomized tamoxifen prevention trials. J Clin Oncol.

[37] Dierssen-Sotos T, Gomez-Acebo I, Palazuelos C, Fernandez-Navarro P, Altzibar JM, Gonzalez-Donquiles C, Ardanaz E, Bustamante M, Alonso-Molero J, Vidal C (2020). Author correction: validating a breast cancer score in Spanish women. The MCC-Spain study. Sci Rep.

[38] Nielsen MA (2015). Neural networks and deep learning.

[39] Sapkota Y, Mackey JR, Lai R, Franco-Villalobos C, Lupichuk S, Robson PJ, Kopciuk K, Cass CE, Yasui Y, Damaraju S (2014). Assessing SNP-SNP interactions among DNA repair, modification and metabolism related pathway genes in breast cancer susceptibility. PLoS One.

[40] Behravan H, Hartikainen JM, Tengstrom M, Kosma VM, Mannermaa A (2020). Predicting breast cancer risk using interacting genetic and demographic factors and machine learning. Sci Rep.

[41] Behravan H, Hartikainen JM, Tengstrom M, Pylkas K, Winqvist R, Kosma VM, Mannermaa A (2018). Machine learning identifies interacting genetic variants contributing to breast cancer risk: a case study in Finnish cases and controls. Sci Rep.

