# Supplementary material for: Development and validation of polygenic risk scores for prediction of breast cancer and breast cancer subtypes in Chinese women
Source: BMC Cancer. 2022 Apr 8;22:374. doi: 10.1186/s12885-022-09425-3 (PMC8991589; doi:10.1186/s12885-022-09425-3)
Supplement: Supplementary file 1 — Additional file 1: Table S1. Basic information of the first set of SNPs along with the SNP selection results. Table S2. Basic information of the second set of SNPs along with SNP selection results. Table S3. Basic information of the 24 SNPs included for PRSs construction. Table S4. Characteristics of the participants in the SBCGS. Table S5 Comparison between the participants in the SBCCS who were included in the current study with the participants excluded due to unavailability of DNA samples or number of failed SNPs ≥3. Table S6. Predictive performance of the primary PRSs for overall and ER+/ER- breast cancer. Table S7. Sensitivity analysis 1 - predictive performance of the primary PRSs for overall and ER+/ER- breast cancer when samples with missing genotypes were all excluded. Table S8 Sensitivity analysis 2 - predictive performance of the SNP-17 PRSRLR for overall and ER+/ER- breast cancer. Table S9. Indirect comparison with previous PRSs for Chinese women. Fig. S1. Linkage disequilibrium matrix of the first set of SNPs. Fig. S2. Linkage disequilibrium matrix of the second set of SNPs. Fig. S3. Hyperparameters tuning results of the logistic ridge regression model. Fig. S4. The structure of the ANN model used in the current study. [file 12885_2022_9425_MOESM1_ESM.docx]

**Supplementary Information For**

**Development and validation of polygenic risk scores for prediction of breast cancer and breast cancer subtypes in Chinese women**

**Table of contents**

[**Table S1** Basic information of the first set of SNPs along with the SNP selection results 2](#_Toc98146875)

[**Table S2** Basic information of the second set of SNPs along with SNP selection results 6](#_Toc98146876)

[**Table S3** Basic information of the 24 SNPs included for PRSs construction 7](#_Toc98146877)

[**Table S4** Characteristics of the participants in the SBCGS 9](#_Toc98146878)

[**Table S5** Comparison between the participants in the SBCCS who were included in the current study with the participants excluded due to unavailability of DNA samples or number of failed SNPs ≥ 3 10](#_Toc98146879)

[**Table S6** Predictive performance of the primary PRSs for overall and ER+/ER- breast cancer 11](#_Toc98146880)

[**Table S7** Sensitivity analysis 1 - predictive performance of the primary PRSs for overall and ER+/ER- breast cancer when samples with missing genotypes were all excluded 13](#_Toc98146881)

[**Table S8** Sensitivity analysis 2 - predictive performance of the SNP-17 PRS_RLR_ for overall and ER+/ER- breast cancer 15](#_Toc98146882)

[**Table S9** Indirect comparison with previous PRSs for Chinese women 16](#_Toc98146883)

[**Figure S1** Linkage disequilibrium matrix of the first set of SNPs 18](#_Toc98146884)

[**Figure S2** Linkage disequilibrium matrix of the second set of SNPs 19](#_Toc98146885)

[**Figure S3** Hyperparameters tuning results of the logistic ridge regression model 20](#_Toc98146886)

[**Figure S4** The structure of the ANN model used in the current study 21](#_Toc98146887)

**Table S1** Basic information of the first set of SNPs along with the SNP selection results

| **SNP** | **Position (GRCh37)** | **Proxy** | **Position for proxy (GRCh37)** | **Included for genotype in the SBCCS (reason for exclusion)** | **N_Missing (N=807)** | **HWE-P (case)** | **HWE-P**  **(control)** | **Included in PRS (reason for exclusion)** |
| --- | --- | --- | --- | --- | --- | --- | --- | --- |
| rs1801133^1^ | chr1:11856378 | - | - | No (no proxy found) | - | - | - | - |
| rs1137100^2^ | chr1:66036441 | - | - | Yes | 3 | 0.40 | 0.90 | Yes |
| rs1137101^3^ | chr1:66058513 | rs10789190 | chr1:66063580 | Yes | 3 | 0.79 | 0.87 | Yes |
| rs763110^4,5^ | chr1:172627498 | - | - | Yes | 3 | 0.99 | 1.00 | Yes |
| rs4973768^6^ | chr3:27416013 | - | - | No (no proxy found) | - | - | - | - |
| rs10941679^7^ | chr5:44706498 | rs4479849 | chr5:44644006 | Yes | 3 | 0.71 | 0.09 | Yes |
| rs9485372^8^ | chr6:149608874 | - | - | Yes | 5 | 0.81 | 1.00 | Yes |
| rs2046210^9^ | chr6:151948366 | - | - | Yes | 0 | 0.75 | 0.10 | Yes |
| rs9397437^9,10^ | chr6:151952332 | - | - | Yes | 2 | 0.06 | 0.23 | Yes |
| rs662^11,12^ | chr7:94937446 | rs2057681 | chr7:94938257 | Yes | 4 | 0.74 | 0.53 | Yes |
| rs854560^11^ | chr7:94946084 | - | - | No (no proxy found) | - | - | - | - |
| rs7799039^3^ | chr7:127878783 | - | - | Yes | 13 | 0.99 | 0.64 | Yes |
| rs2234767^13,14^ | chr10:90749256 | rs7097467 | chr10:90753244 | Yes | 3 | 0.67 | 0.98 | Yes |
| rs2981578^15,16^ | chr10:123340311 | rs10736303 | chr10:123334457 | Yes | 2 | 0.99 | 0.98 | Yes |
| rs2981575^16^ | chr10:123346116 | - | - | Yes | 16 | 0.82 | 0.89 | Yes |
| rs1219648^16,17^ | chr10:123346190 | - | - | No (in LD with rs2162540 and rs2981575) | - | - | - | - |
| rs2420946^16,17^ | chr10:123351324 | rs2162540 | chr10:123352136 | Yes | 5 | 0.97 | 0.67 | Yes |
| rs2981582^15,16^ | chr10:123352317 | - | - | Yes | 1 | 0.99 | 0.98 | Yes |
| rs3817198^18^ | chr11:1909006 | - | - | Yes | 5 | 0.73 | 0.62 | Yes |
| rs1695^19^ | chr11:67352689 | - | - | No (no proxy found) | - | - | - | - |
| rs730154^20^ | chr15:51591204 | rs8031463 | chr15:51596539 | Yes | 0 | 1.00 | 0.67 | Yes |
| rs9282861^21^ | chr16:28617514 | - | - | No (no proxy found) | - | - | - | - |
| rs8051542^22^ | chr16:52534167 | - | - | Yes | 0 | 0.98 | 0.99 | Yes |
| rs3803662^22,23^ | chr16:52586341 | - | - | Yes | 4 | 0.85 | 0.20 | Yes |
| rs4784227^24^ | chr16:52599188 | - | - | Yes | 0 | 0.85 | 0.92 | Yes |
| rs12922061^23^ | chr16:52635000 | - | - | Yes | 3 | 0.88 | 0.56 | Yes |
| rs11655505^25^ | chr17:41278377 | rs9646413 | chr17:41426157 | Yes | 0 | 0.18 | 0.70 | Yes |
| rs4680^26^ | chr22:19951271 | - | - | Yes | 0 | 1.00 | 0.83 | Yes |
| Eligible proxy needs to be in tight LD (R^2^ > 0.9) with the original SNP; 22 SNPs from the first set of SNPs were genotyped in the SBCCS samples (N = 807).  HWE-P: P-value for Hardy Weinberg equilibrium test. | | | | | | | | |

References:

1. Yu L, Chen J. Association of MHTFR Ala222Val (rs1801133) polymorphism and breast cancer susceptibility: An update meta-analysis based on 51 research studies. Diagnostic pathology. 2012;7(1):171.

2. Wang L-q, Shen W, Xu L, et al. The association between polymorphisms in the leptin receptor gene and risk of breast cancer: a systematic review and pooled analysis. Breast cancer research and treatment. 2012;136(1):231-239.

3. Luan H, Zhang H, Li Y, et al. Association of two obesity-related gene polymorphisms LEPG2548A rs7799039 and LEPRQ223R rs1137101 with the risk of breast cancer. Oncotarget. 2017;8(35):59333.

4. Wang M, Wang Z, Wang X-J, et al. Distinct role of the Fas rs1800682 and FasL rs763110 polymorphisms in determining the risk of breast cancer among Han Chinese females. Drug design, development and therapy. 2016;10:2359.

5. Wang Z, Gu J, Nie W, Xu J, Huang G, Guan X. Quantitative assessment of the association between three polymorphisms in FAS and FASL gene and breast cancer risk. Tumor Biology. 2014;35(4):3035-3039.

6. Chen W, Zhong R, Ming J, et al. The SLC4A7 variant rs4973768 is associated with breast cancer risk: evidence from a case–control study and a meta-analysis. Breast cancer research and treatment. 2012;136(3):847-857.

7. Wang X, Zhang L, Chen Z, et al. Association between 5p12 genomic markers and breast cancer susceptibility: evidence from 19 case-control studies. PloS one. 2013;8(9):e73611.

8. Long J, Cai Q, Sung H, et al. Genome-wide association study in east Asians identifies novel susceptibility loci for breast cancer. PLoS genetics. 2012;8(2):e1002532.

9. Zheng W, Long J, Gao Y-T, et al. Genome-wide association study identifies a new breast cancer susceptibility locus at 6q25. 1. Nature genetics. 2009;41(3):324-328.

10. Hirata T, Koga K, Johnson TA, et al. Japanese GWAS identifies variants for bust-size, dysmenorrhea, and menstrual fever that are eQTLs for relevant protein-coding or long non-coding RNAs. Scientific reports. 2018;8(1):1-17.

11. Liu P, Wang Q, Cui Y, Wang J. A meta-analysis of the relationship between paraoxonase 1 polymorphisms and cancer. Free Radical Research. 2019;53(11-12):1045-1050.

12. Saadat M. Paraoxonase 1 genetic polymorphisms and susceptibility to breast cancer: a meta-analysis. Cancer Epidemiology. 2012;36(2):e101-e103.

13. Wang W, Zheng Z, Yu W, Lin H, Cui B, Cao F. Polymorphisms of the FAS and FASL genes and risk of breast cancer. Oncology letters. 2012;3(3):625-628.

14. Chen Y, Wang H, Yan Y, Ren M, Yan C, Wang B. Correlation between FAS single nucleotide polymorphisms and breast carcinoma susceptibility in Asia. Medicine. 2019;98(49).

15. Zhang Y, Zeng X, Liu P, et al. Association between FGFR2 (rs2981582, rs2420946 and rs2981578) polymorphism and breast cancer susceptibility: a meta-analysis. Oncotarget. 2017;8(2):3454.

16. Cui F, Wu D, Wang W, He X, Wang M. Variants of FGFR2 and their associations with breast cancer risk: a HUGE systematic review and meta-analysis. Breast cancer research and treatment. 2016;155(2):313-335.

17. Zhang Y, Lu H, Ji H, et al. Association between rs11200014, rs2981579, and rs1219648 polymorphism and breast cancer susceptibility: A meta-analysis. Medicine. 2017;96(50).

18. Tang J, Li H, Luo J, Mei H, Peng L, Li X. The LSP1 rs3817198 T> C polymorphism contributes to increased breast cancer risk: a meta-analysis of twelve studies. Oncotarget. 2016;7(39):63960.

19. Kuang M, Xu W, Cao C, et al. Glutathione S-transferase P1 rs1695 A> G polymorphism and breast cancer risk: evidence from a meta-analysis. Genet Mol Res. 2016;15(2).

20. Wang Q, Li H, Tao P, et al. Soy isoflavones, CYP1A1, CYP1B1, and COMT polymorphisms, and breast cancer: a case–control study in southwestern China. DNA and cell biology. 2011;30(8):585-595.

21. Forat-Yazdi M, Jafari M, Kargar S, et al. Association between SULT1A1 Arg213His (Rs9282861) polymorphism and risk of breast cancer: A systematic review and meta-analysis. Journal of Research in Health Sciences. 2017;17(4):396.

22. Zhang L, Long X. Association of three SNPs in TOX3 and breast cancer risk: Evidence from 97275 cases and 128686 controls. Scientific reports. 2015;5:12773.

23. Liao J, Chen Y, Zhu J, Wang Q, Mo Z. Polymorphisms in the TOX3/LOC643714 and risk of breast cancer in south China. The International Journal of Biological Markers. 2018;33(4):492-499.

24. Sun Y, Chen P, Wu J, et al. Association of polymorphisms in LOC105377871 and CASC16 with breast cancer in the northwest Chinese Han population. The Journal of Gene Medicine. 2020;22(3):e3131.

25. Chan KY, Liu W, Long J-R, et al. Functional polymorphisms in the BRCA1 promoter influence transcription and are associated with decreased risk for breast cancer in Chinese women. Journal of medical genetics. 2009;46(1):32-39.

26. Qiu J, Du Z, Liu J, Zhou Y, Liang F, Lü Q. Association between polymorphisms in estrogen metabolism genes and breast cancer development in Chinese women: A prospective case–control study. Medicine. 2018;97(47).

**Table S2** Basic information of the second set of SNPs along with SNP selection results

| **SNP** | **Position (GRCh37)** | **Effect allele** | **Training set (SBCGS)** | | | **Test set (SBCCS)** | | | |  |  |
| --- | --- | --- | --- | --- | --- | --- | --- | --- | --- | --- | --- |
|  |  |  | **OR** | **P-value** | **Included for genotype** | **OR** | **P-value** | **HWE-P**  **(case)** | **HWE-P**  **(control)** | **N_Missing (N=807)** | **Included in PRS** |
| rs6730484 | chr2:79157621 | A | 0.60 | 3.60E-08 | Yes | 1.86 | 0.01 | <0.001 | <0.001 | 5 | No |
| rs1440625 | chr3:74159454 | C | 0.62 | 5.25E-09 | Yes | 1.04 | 0.90 | 0.85 | 0.86 | 3 | No |
| rs1583563 | chr3:161556214 | G | 0.52 | 1.64E-15 | Yes | 3.59 | 0.05 | 0.96 | 1.00 | 3 | No |
| rs13113498 | chr4:125011101 | A | 0.61 | 1.31E-08 | Yes | 1.26 | 0.51 | 0.89 | 0.93 | 1 | No |
| rs9477834 | chr6:18713337 | A | 0.47 | 1.55E-15 | Yes | - | 1.00 | - | 1.00 | 3 | No |
| rs11773166 | chr7:86040733 | C | 0.76 | 1.28E-08 | Yes | 1.38 | 0.01 | 0.98 | 0.96 | 1 | No |
| rs7863754 | chr9:20168739 | T | 0.62 | 1.58E-08 | Yes | 0.78 | 0.25 | 0.60 | 0.90 | 3 | Yes |
| rs292433 | chr11:30736383 | A | 0.57 | 5.66E-10 | Yes | 1.33 | 0.42 | 0.87 | 0.93 | 2 | No |
| rs7120385 | chr11:106507197 | T | 0.64 | 6.54E-09 | Yes | 1.09 | 0.75 | 0.17 | 0.79 | 5 | No |
| rs41464146 | chr13:47764748 | C | 1.34 | 9.20E-10 | Yes | 0.96 | 0.82 | 1.00 | 0.47 | 3 | No |
| rs1417216 | chr13:66304003 | A | 0.51 | 8.08E-13 | Yes | 0.68 | 0.45 | 0.99 | 0.97 | 3 | Yes |
| rs9323935 | chr14:27717045 | A | 0.59 | 1.22E-10 | Yes | 1.93 | 0.19 | 0.95 | 0.99 | 3 | No |
| These SNPs were selected to be genotyped in the SBCCS samples because they achieved genome-wide significance (P < 5×10^-8^) for overall breast cancer on the SBCGS dataset; ten SNPs were excluded from further analysis because they had opposite effects on breast cancer in the SBCGS and SBCCS.  HWE-P: P-value for Hardy Weinberg equilibrium test. | | | | | | | | | | | |

**Table S3** Basic information of the 24 SNPs included for PRSs construction

| **SNP** | **Position**  **(GRCh37)** | **Effect allele** | **Gene** | **Training set (SBCGS)** | | **Test set (SBCCS, N=801)** | | |
| --- | --- | --- | --- | --- | --- | --- | --- | --- |
|  |  |  |  | **MAF** | **OR** | **N_Missing** | **MAF** | **OR** |
| rs1137100 | chr1:66036441 | A | LEPR | 0.16 | 1.08 | 0 | 0.17 | 1.23 |
| rs10789190 | chr1:66063580 | G | LEPR | 0.12 | 1.15 | 0 | 0.12 | 1.08 |
| rs763110 | chr1:172627498 | T | FASLG | 0.25 | 1.07 | 0 | 0.26 | 1.12 |
| rs4479849 | chr5:44644006 | G | Unknow | 0.49 | 0.98 | 2 | 0.50 | 1.01 |
| rs9485372 | chr6:149608874 | A | TAB2 | 0.41 | 0.88 | 2 | 0.41 | 0.83 |
| rs2046210 | chr6:151948366 | A | ESR1 | 0.39 | 1.24 | 0 | 0.40 | 1.28 |
| rs9397437 | chr6:151952332 | A | ESR1 | 0.34 | 1.26 | 1 | 0.34 | 1.44 |
| rs2057681 | chr7:94938257 | A | PON1 | 0.37 | 1.03 | 2 | 0.35 | 1.03 |
| rs7799039 | chr7:127878783 | G | LEP | 0.25 | 1.07 | 11 | 0.23 | 1.15 |
| rs7863754 | chr9:20168739 | T | Unknow | 0.07 | 0.62 | 0 | 0.06 | 0.78 |
| rs7097467 | chr10:90753244 | C | FAS | 0.36 | 0.94 | 2 | 0.39 | 1.06 |
| rs10736303 | chr10:123334457 | A | FGFR2 | 0.42 | 0.83 | 0 | 0.44 | 0.80 |
| rs2981575 | chr10:123346116 | G | FGFR2 | 0.45 | 1.15 | 14 | 0.46 | 1.18 |
| rs2162540 | chr10:123352136 | C | FGFR2 | 0.41 | 1.12 | 3 | 0.41 | 1.17 |
| rs2981582 | chr10:123352317 | A | FGFR2 | 0.34 | 1.15 | 0 | 0.35 | 1.11 |
| rs3817198 | chr11:1909006 | C | LSP1 | 0.13 | 1.06 | 2 | 0.10 | 1.19 |
| rs1417216 | chr13:66304003 | A | Unknow | 0.05 | 0.51 | 0 | 0.01 | 0.68 |
| rs8031463 | chr15:51596539 | C | CYP19 | 0.34 | 0.94 | 0 | 0.32 | 0.93 |
| rs8051542 | chr16:52534167 | T | TOX3 | 0.19 | 1.07 | 0 | 0.19 | 1.03 |
| rs3803662 | chr16:52586341 | G | TOX3 | 0.34 | 0.93 | 1 | 0.37 | 1.11 |
| rs4784227 | chr16:52599188 | T | TOX3 | 0.27 | 1.20 | 0 | 0.26 | 1.09 |
| rs12922061 | chr16:52635000 | T | CASC16 | 0.27 | 1.16 | 0 | 0.26 | 1.06 |
| rs9646413 | chr17:41426157 | G | BRCA1 | 0.37 | 1.06 | 0 | 0.37 | 1.09 |
| rs4680 | chr22:19951271 | A | COMT | 0.27 | 0.97 | 0 | 0.26 | 0.95 |
| Gene: gene that the SNP is located or its nearby gene; MAF: minor allele frequency; | | | | | | | | |

**Table S4** Characteristics of the participants in the SBCGS

| **Characteristics** | **Controls**  **(N=2,139)** | **Cases**  **(N=2,722)** | **P-value**^*^ |
| --- | --- | --- | --- |
| **Age** | | | |
| 20-35 | 44（2.1%） | 47（1.7%） | <0.001 |
| 36-50 | 1,150（53.8%） | 1,381（50.7%） |  |
| 51-65 | 824（38.5%） | 1,032（37.9%） |  |
| 66-80 | 121（56.6%） | 262（9.6%） |  |
| **ER status** | | | |
| Positive | NA | 985（65.9%） | NA |
| Negative |  | 510（34.1%） |  |
| Missing |  | 1227 |  |
| ^*^P-value from Pearson chi-squared test | | | |

**Table S5** Comparison between the participants in the SBCCS who were included in the current study with the participants excluded due to unavailability of DNA samples or number of failed SNPs ≥ 3

| **Characteristics** | **Controls excluded (N=367)** | **Controls included (N=427)** | **P-value*** | **Cases excluded (N=431)** | **Cases included (N=374)** | **P-value*** |
| --- | --- | --- | --- | --- | --- | --- |
| **Continuous variables (median, IQR)** | |  |  |  |  |  |
| Age (years) | 50.00 (43.00-57.00) | 50.00 (44.00-57.00) | 0.32 | 48.00 (43.00-55.00) | 48.00 (42.00-53.00) | 0.26 |
| BMI (kg/m2) | 22.67 (20.83-24.95) | 22.94 (21.23-25.24) | 0.12 | 22.89 (21.09-25.21) | 23.37 (21.46-25.10) | 0.11 |
| Age at menarche (years) | 14.00 (13.00-15.00) | 14.00 (13.00-15.00) | 0.32 | 14.00 (13.00-15.00) | 14.00 (13.00-15.00) | 0.36 |
| Number of live births (N) | 1.00 (1.00-2.00) | 1.00 (1.00-2.00) | 0.15 | 1.00 (1.00-1.00) | 1.00 (1.00-1.00) | 0.09 |
| Gail-2 model 5-year risk (%) | 0.57 (0.46-0.68) | 0.54 (0.46-0.67) | 0.49 | 0.50 (0.46-0.67) | 0.54 (0.42-0.67) | 0.29 |
| **Categorical variables (N, %)** | |  |  |  |  |  |
| Menopausal status |  |  | 0.84 |  |  | 0.29 |
| Premenopausal | 209 (56.95%) | 239 (55.97%) |  | 240 (55.68%) | 223 (59.63%) |  |
| Postmenopausal | 158 (43.05%) | 188 (44.03%) |  | 191 (44.32%) | 151 (40.37%) |  |
| Family history of breast cancer |  |  | 0.90 |  |  | 0.84 |
| Yes | 9 (2.45%) | 10 (2.34%) |  | 10 (2.32%) | 7 (1.87%) |  |
| No | 358 (97.55%) | 417 (97.66%) |  | 421 (97.68%) | 367 (98.13%) |  |
| ^*^P-value from Mann-Whitney U test (continuous variables) or chi-squared test (categorical variables) | | | | | | |

**Table S6** Predictive performance of the primary PRSs for overall and ER+/ER- breast cancer

| **PRS** | **N (Controls/Cases)** | **Cases (Median, IQR)** | **Controls (Median, IQR)** | **Q_4th_ vs Q_1st_ OR (95% CI)** | **IQ-OR (95% CI)** | **O/E OR  (95% CI)** | **AUC (95% CI)** | **Adjustment^*^** |
| --- | --- | --- | --- | --- | --- | --- | --- | --- |
| **All breast cancer cases** | |  |  |  |  |  |  |  |
| PRS_RLR_ | 374/427 | 0.62 (0.23-1.05) | 0.44 (0.05-0.84) | 2.09 (1.40-3.11) | 1.49 (1.23-1.81) | 1.10 (0.71-1.48) | 0.586 (0.547-0.625) | None |
|  |  | 0.17 (-0.22-0.6) | -0.01 (-0.41-0.39) | 2.18 (1.46-3.26) | 1.49 (1.23-1.82) | 1.07 (0.63-1.51) | 0.586 (0.547-0.625) | A |
| PRS_LRR_ |  | 0.14 (-0.06-0.32) | 0.02 (-0.18-0.23) | 2.59 (1.71-3.91) | 1.58 (1.29-1.92) | 1.08 (0.62-1.55) | 0.598 (0.559-0.637) | None |
|  |  | 0.11 (-0.09-0.29) | -0.01 (-0.21-0.2) | 2.40 (1.60-3.61) | 1.57 (1.29-1.91) | 1.08 (0.58-1.58) | 0.598 (0.559-0.637) | A |
| PRS_ANN_ |  | 0.01 (-0.24-0.13) | -0.17 (-0.33-0.09) | 2.61 (1.72-3.95) | 1.76 (1.39-2.24) | 1.09 (0.77-1.41) | 0.601 (0.562-0.640) | None |
|  |  | 0.15 (-0.1-0.27) | -0.02 (-0.19-0.23) | 2.81 (1.85-4.28) | 1.75 (1.38-2.21) | 1.13 (0.83-1.42) | 0.600 (0.561-0.639) | A |
| **ER+ breast cancer cases** | |  |  |  |  |  |  |  |
| PRS_RLR_ | 374/290 | 0.65 (0.24-1.06) | 0.44 (0.05-0.84) | 2.24 (1.44-3.48) | 1.56 (1.26-1.93) | 1.09 (0.61-1.57) | 0.597 (0.553-0.641) | None |
|  |  | 0.19 (-0.21-0.62) | -0.01 (-0.41-0.39) | 2.37 (1.51-3.72) | 1.56 (1.26-1.94) | 1.05 (0.56-1.53) | 0.596 (0.552-0.640) | A |
| PRS_LRR_ |  | 0.15 (-0.05-0.33) | 0.02 (-0.18-0.23) | 2.94 (1.85-4.69) | 1.67 (1.34-2.08) | 1.12 (0.74-1.51) | 0.613 (0.570-0.656) | None |
|  |  | 0.12 (-0.07-0.3) | -0.01 (-0.21-0.2) | 2.69 (1.70-4.26) | 1.66 (1.34-2.07) | 1.10 (0.61-1.59) | 0.612 (0.569-0.655) | A |
| PRS_ANN_ |  | 0.04 (-0.22-0.15) | -0.17 (-0.33-0.09) | 3.00 (1.87-4.78) | 1.96 (1.50-2.55) | 1.09 (0.80-1.38) | 0.620 (0.577-0.663) | None |
|  |  | 0.18 (-0.09-0.29) | -0.02 (-0.19-0.23) | 3.25 (2.03-5.23) | 1.93 (1.48-2.52) | 1.11 (0.74-1.48) | 0.619 (0.576-0.662) | A |
| **ER- breast cancer cases** | |  |  |  |  |  |  |  |
| PRS_RLR_ | 374/124 | 0.57 (0.19-0.92) | 0.44 (0.05-0.84) | 1.63 (0.91-2.95) | 1.27 (0.96-1.69) | 1.08 (0.42-1.74) | 0.554 (0.495-0.613) | None |
|  |  | 0.13 (-0.27-0.46) | -0.01 (-0.41-0.39) | 1.63 (0.91-2.95) | 1.28 (0.96-1.70) | 1.00 (0.26-1.75) | 0.553 (0.494-0.612) | A |
| PRS_LRR_ |  | 0.08 (-0.11-0.28) | 0.02 (-0.18-0.23) | 1.79 (0.98-3.28) | 1.29 (0.97-1.72) | 1.23 (0.18-2.28) | 0.555 (0.496-0.614) | None |
|  |  | 0.05 (-0.15-0.25) | -0.01 (-0.21-0.2) | 1.72 (0.95-3.11) | 1.29 (0.97-1.71) | 1.11 (0.00-2.22) | 0.556 (0.497-0.615) | A |
| PRS_ANN_ |  | -0.13 (-0.25-0.11) | -0.17 (-0.33-0.09) | 1.78 (0.96-3.30) | 1.32 (0.93-1.87) | 1.37 (-0.62-3.35) | 0.550 (0.491-0.609) | None |
|  |  | 0.01 (-0.12-0.24) | -0.02 (-0.19-0.23) | 1.90 (1.02-3.52) | 1.32 (0.93-1.86) | 1.40 (-0.42-3.23) | 0.550 (0.491-0.609) | A |
| ^*^Adjustment A: adjusted for Gail-2 model 5-year absolute risk.  ER^+^: estrogen receptor positive; ER^-^: estrogen receptor negative; OR: odds ratio; Q_4th_: fourth quartile; Q_1st_: first quartile; IQR: interquartile range; PRS: polygenic risk score; RLR: repeated logistic regression; LRR: logistic ridge regression; ANN: Artificial Neural Network; IQ-OR: OR per IQR increase of the PRS in controls; O/E OR: observed to expected OR; AUC: area under the receiver operator characteristic curve. | | | | | | | | |

**Table S7** Sensitivity analysis 1 - predictive performance of the primary PRSs for overall and ER+/ER- breast cancer when samples with missing genotypes were all excluded

| **PRS** | **N (Controls/Cases)** | **Cases (Median, IQR)** | **Controls (Median, IQR)** | **Q_4th_ vs Q_1st_ OR (95% CI)** | **IQ-OR (95% CI)** | **O/E OR  (95% CI)** | **AUC (95% CI)** | **Adjustment^*^** |
| --- | --- | --- | --- | --- | --- | --- | --- | --- |
| **All breast cancer cases** | |  |  |  |  |  |  |  |
| PRS_RLR_ | 358/405 | 0.62 (0.23-1.06) | 0.45 (0.05-0.85) | 2.2 (1.46-3.31) | 1.49 (1.22-1.82) | 1.1 (0.67-1.54) | 0.586 (0.546-0.626) | None |
|  |  | 0.17 (-0.23-0.59) | -0.01 (-0.41-0.39) | 2.28 (1.51-3.45) | 1.49 (1.22-1.82) | 1.08 (0.59-1.57) | 0.586 (0.546-0.626) | A |
|  |  | 0.16 (-0.24-0.58) | -0.01 (-0.4-0.38) | 2.0 (1.33-3.0) | 1.44 (1.19-1.75) | 1.09 (0.55-1.64) | 0.582 (0.542-0.622) | B |
| PRS_LRR_ |  | 0.14 (-0.05-0.32) | 0.02 (-0.18-0.23) | 2.54 (1.66-3.88) | 1.57 (1.28-1.92) | 1.09 (0.61-1.57) | 0.598 (0.558-0.638) | None |
|  |  | 0.1 (-0.09-0.29) | -0.01 (-0.21-0.2) | 2.52 (1.65-3.85) | 1.57 (1.28-1.93) | 1.07 (0.61-1.53) | 0.598 (0.558-0.638) | A |
|  |  | 0.1 (-0.1-0.29) | -0.0 (-0.21-0.22) | 2.24 (1.47-3.4) | 1.57 (1.27-1.94) | 1.06 (0.72-1.41) | 0.594 (0.554-0.634) | B |
| PRS_ANN_ |  | 0.01 (-0.24-0.13) | -0.16 (-0.33-0.09) | 2.52 (1.65-3.86) | 1.76 (1.38-2.24) | 1.06 (0.74-1.38) | 0.601 (0.561-0.641) | None |
|  |  | 0.15 (-0.1-0.27) | -0.02 (-0.19-0.23) | 2.59 (1.69-3.97) | 1.76 (1.38-2.24) | 1.06 (0.8-1.32) | 0.6 (0.56-0.64) | A |
|  |  | 0.15 (-0.1-0.27) | -0.02 (-0.19-0.22) | 2.42 (1.6-3.66) | 1.69 (1.33-2.14) | 1.14 (0.59-1.69) | 0.596 (0.556-0.636) | B |
| **ER+ breast cancer cases** | |  |  |  |  |  |  |  |
| PRS_RLR_ | 358/274 | 0.64 (0.24-1.06) | 0.45 (0.05-0.85) | 2.42 (1.53-3.83) | 1.56 (1.25-1.95) | 1.12 (0.6-1.64) | 0.596 (0.551-0.641) | None |
|  |  | 0.18 (-0.22-0.6) | -0.01 (-0.41-0.39) | 2.56 (1.61-4.07) | 1.57 (1.25-1.95) | 1.11 (0.48-1.74) | 0.596 (0.551-0.641) | A |
|  |  | 0.18 (-0.21-0.6) | -0.01 (-0.4-0.38) | 2.23 (1.42-3.51) | 1.52 (1.22-1.88) | 1.12 (0.37-1.86) | 0.593 (0.548-0.638) | B |
| PRS_LRR_ |  | 0.15 (-0.05-0.33) | 0.02 (-0.18-0.23) | 2.96 (1.83-4.79) | 1.67 (1.34-2.1) | 1.1 (0.71-1.49) | 0.612 (0.568-0.656) | None |
|  |  | 0.12 (-0.07-0.3) | -0.01 (-0.21-0.2) | 2.93 (1.81-4.75) | 1.68 (1.34-2.1) | 1.11 (0.76-1.47) | 0.612 (0.568-0.656) | A |
|  |  | 0.13 (-0.08-0.3) | -0.0 (-0.21-0.22) | 2.57 (1.6-4.13) | 1.68 (1.33-2.13) | 1.08 (0.75-1.4) | 0.608 (0.563-0.653) | B |
| PRS_ANN_ |  | 0.04 (-0.22-0.15) | -0.16 (-0.33-0.09) | 2.93 (1.81-4.74) | 1.96 (1.49-2.58) | 1.07 (0.75-1.38) | 0.62 (0.576-0.664) | None |
|  |  | 0.18 (-0.08-0.29) | -0.02 (-0.19-0.23) | 2.97 (1.83-4.82) | 1.96 (1.49-2.57) | 1.08 (0.78-1.38) | 0.62 (0.576-0.664) | A |
|  |  | 0.17 (-0.09-0.28) | -0.02 (-0.19-0.22) | 2.82 (1.77-4.5) | 1.87 (1.43-2.44) | 1.11 (0.59-1.64) | 0.615 (0.571-0.659) | B |
| **ER- breast cancer cases** | |  |  |  |  |  |  |  |
| PRS_RLR_ | 358/118 | 0.59 (0.18-0.92) | 0.45 (0.05-0.85) | 1.65 (0.91-2.99) | 1.28 (0.95-1.71) | 1.11 (0.48-1.74) | 0.553 (0.492-0.614) | None |
|  |  | 0.13 (-0.27-0.46) | -0.01 (-0.41-0.39) | 1.65 (0.9-2.99) | 1.28 (0.95-1.71) | 1.15 (0.72-1.58) | 0.553 (0.492-0.614) | A |
|  |  | 0.1 (-0.29-0.5) | -0.01 (-0.4-0.38) | 1.4 (0.77-2.53) | 1.24 (0.93-1.64) | 1.11 (0.15-2.07) | 0.548 (0.487-0.609) | B |
| PRS_LRR_ |  | 0.09 (-0.12-0.28) | 0.02 (-0.18-0.23) | 1.68 (0.91-3.09) | 1.29 (0.96-1.73) | 1.15 (0.16-2.15) | 0.555 (0.494-0.616) | None |
|  |  | 0.06 (-0.15-0.25) | -0.01 (-0.21-0.2) | 1.68 (0.91-3.09) | 1.29 (0.96-1.73) | 1.18 (0.34-2.02) | 0.555 (0.494-0.616) | A |
|  |  | 0.06 (-0.13-0.25) | -0.0 (-0.21-0.22) | 1.57 (0.85-2.9) | 1.28 (0.94-1.75) | 1.11 (0.21-2.01) | 0.552 (0.491-0.613) | B |
| PRS_ANN_ |  | -0.11 (-0.27-0.11) | -0.16 (-0.33-0.09) | 1.69 (0.91-3.15) | 1.32 (0.93-1.88) | 1.33 (-0.5-3.15) | 0.549 (0.488-0.61) | None |
|  |  | 0.03 (-0.13-0.25) | -0.02 (-0.19-0.23) | 1.8 (0.97-3.35) | 1.32 (0.93-1.88) | 1.47 (-0.43-3.37) | 0.549 (0.488-0.61) | A |
|  |  | 0.0 (-0.11-0.23) | -0.02 (-0.19-0.22) | 1.67 (0.9-3.08) | 1.29 (0.91-1.82) | 1.04 (-0.29-2.37) | 0.546 (0.485-0.607) | B |
| ^*^Adjustment A: adjusted for Gail-2 model 5-year absolute risk; adjustment B: adjusted for classical breast cancer risk factors.  ER^+^: estrogen receptor positive; ER^-^: estrogen receptor negative; OR: odds ratio; Q_4th_: fourth quartile; Q_1st_: first quartile; IQR: interquartile range; PRS: polygenic risk score; RLR: repeated logistic regression; LRR: logistic ridge regression; ANN: Artificial Neural Network; IQ-OR: OR per IQR increase of the PRS in controls; O/E OR: observed to expected OR; AUC: area under the receiver operator characteristic curve. | | | | | | | | |

**Table S8** Sensitivity analysis 2 - predictive performance of the SNP-17 PRS_RLR_ for overall and ER+/ER- breast cancer

| **PRS** | **N (Controls/Cases)** | **Cases (Median, IQR)** | **Controls (Median, IQR)** | **Q_4th_ vs Q_1st_ OR (95% CI)** | **IQ-OR (95% CI)** | **O/E OR  (95% CI)** | **AUC (95% CI)** | **Adjustment^*^** |
| --- | --- | --- | --- | --- | --- | --- | --- | --- |
| **All breast cancer cases** | |  |  |  |  |  |  |  |
| PRS_RLR_ | 374/427 | 0.02 (-0.24-0.21) | -0.14 (-0.36-0.1) | 2.34 (1.56-3.51) | 1.53 (1.26-1.85) | 1.07 (0.69-1.45) | 0.596 (0.557-0.635) | None |
|  |  | 0.14 (-0.11-0.34) | 0.0 (-0.22-0.23) | 2.26 (1.52-3.38) | 1.5 (1.25-1.82) | 1.12 (0.64-1.6) | 0.595 (0.556-0.634) | A |
|  |  | 0.13 (-0.11-0.32) | 0.01 (-0.23-0.23) | 2.29 (1.53-3.43) | 1.48 (1.22-1.79) | 1.12 (0.8-1.43) | 0.59 (0.551-0.629) | B |
| **ER+ breast cancer cases** | |  |  |  |  |  |  |  |
| PRS_RLR_ | 374/290 | 0.04 (-0.21-0.23) | -0.14 (-0.36-0.1) | 2.63 (1.68-4.12) | 1.64 (1.32-2.03) | 1.09 (0.72-1.46) | 0.612 (0.569-0.655) | None |
|  |  | 0.17 (-0.09-0.36) | 0.0 (-0.22-0.23) | 2.45 (1.58-3.81) | 1.61 (1.3-1.98) | 1.09 (0.6-1.57) | 0.611 (0.568-0.654) | A |
|  |  | 0.17 (-0.1-0.34) | 0.01 (-0.23-0.23) | 2.62 (1.68-4.09) | 1.58 (1.27-1.95) | 1.09 (0.57-1.6) | 0.605 (0.562-0.648) | B |
| **ER- breast cancer cases** | |  |  |  |  |  |  |  |
| PRS_RLR_ | 374/124 | -0.07 (-0.27-0.11) | -0.14 (-0.36-0.1) | 1.66 (0.89-3.1) | 1.27 (0.96-1.68) | 1.2 (0.01-2.39) | 0.552 (0.493-0.611) | None |
|  |  | 0.05 (-0.15-0.25) | 0.0 (-0.22-0.23) | 1.77 (0.95-3.29) | 1.26 (0.96-1.66) | 1.31 (-0.18-2.8) | 0.552 (0.493-0.611) | A |
|  |  | 0.04 (-0.13-0.25) | 0.01 (-0.23-0.23) | 1.58 (0.86-2.9) | 1.25 (0.94-1.65) | 1.04 (-0.24-2.32) | 0.548 (0.489-0.607) | B |
| ^*^Adjustment A: adjusted for Gail-2 model 5-year absolute risk; adjustment B: adjusted for classical breast cancer risk factors.  ER^+^: estrogen receptor positive; ER^-^: estrogen receptor negative; OR: odds ratio; Q_4th_: fourth quartile; Q_1st_: first quartile; IQR: interquartile range; PRS: polygenic risk score; RLR: repeated logistic regression; LRR: logistic ridge regression; ANN: Artificial Neural Network; IQ-OR: OR per IQR increase of the PRS in controls; O/E OR: observed to expected OR; AUC: area under the receiver operator characteristic curve. | | | | | | | | |

**Table S9** Indirect comparison with previous PRSs for Chinese women

| **Study** | **SNPs** | **Predictive ability** |
| --- | --- | --- |
|  |  |  |
| Zheng et al. | **rs2046210**, **rs1219648**, **rs3817198**, **rs8051542**, **rs3803662**, rs889312, **rs10941679**, rs13281615 | Q_5th_ vs Q_1st_ OR: 1.83 |
| Dai et al. | rs13387042, rs2307032, rs2180341, **rs2046210**, rs2981582 | Q_4th_ vs Q_1st_ OR: 1.91 AUC: 0.573 |
| Lee et al. | rs616488, rs11552449, rs11249433, rs4849887, rs2016394, rs13387042, rs16857609, rs6762644, rs4973768, rs12493607, rs9790517, rs6828523, rs4415084, **rs10941679**, rs889312, rs10472076, rs1432679, rs11242675, rs2180341, rs204247, rs17529111, rs3757318, **rs2046210**, rs720475, rs9693444, rs13281615, rs1562430, rs1011970, rs10759243, rs865686, rs10822013, rs10995190, rs704010, rs1219648, **rs2981582**, **rs3817198**, rs3903072, rs11820646, rs12422552, rs10771399, rs17356907, rs2236007, rs941764, **rs4784227**, rs3112612, rs13329835, rs17817449, rs527616, rs1436904, rs3760982, rs2823093 | Q_4th_ vs Q_1st_ OR: 1.75 |
| Hsieh et al. | **rs2981582**, rs981782, rs889312, **rs3803662**, rs10822013, rs3784099 | Q_4th_ vs Q_1st_ OR: 2.26 AUC: 0.598 |
| Chan et al. | rs16886165, rs3757318, rs11155804, rs12662670, **rs2046210**, rs10816625, rs704010, rs909116, rs7297051, **rs4784227**, rs745570 | Q_4th_ vs Q_1st_ OR: 2.01 AUC: 0.565 |
| Current study (PRS_LRR_ and PRS_ANN_) | See Supplementary Table S3 | Q_4th_ vs Q_1st_ OR: 2.47-2.51 AUC: 0.595-0.596 |
| SNPs that were also included in the current SNP-24 were shown in bold text.  OR: odds ratio; Q_4th_: fourth quartile; Q_1st_: first quartile; IQR: interquartile range; PRS: polygenic risk score; RLR: repeated logistic regression; LRR: logistic ridge regression; ANN: Artificial Neural Network; AUC: area under the receiver operator characteristic curve. | | |

**Figure S1** Linkage disequilibrium matrix of the first set of SNPs


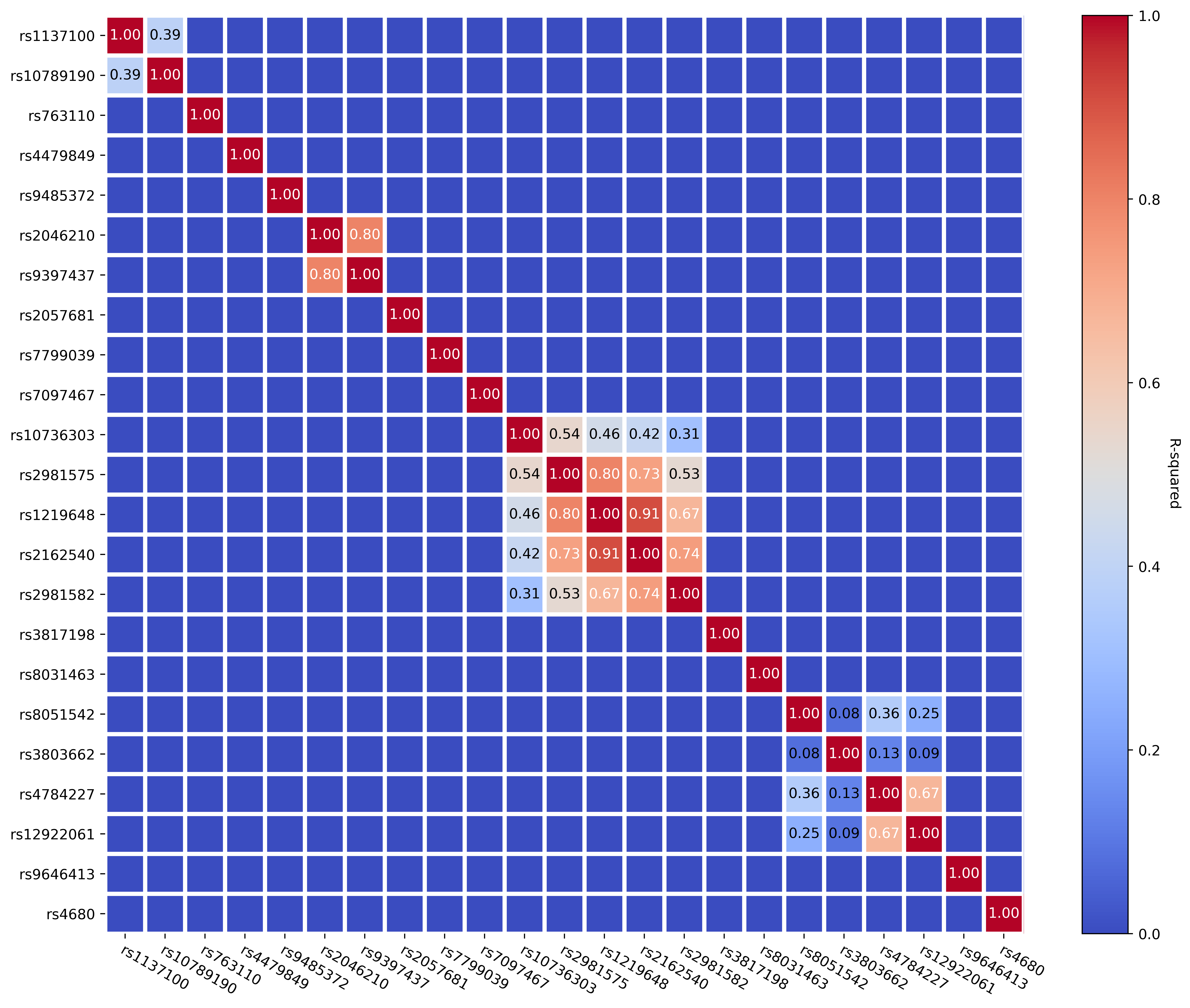


Pairwise R^2^ were calculated based on the SBCGS dataset.

**Figure S2** Linkage disequilibrium matrix of the second set of SNPs


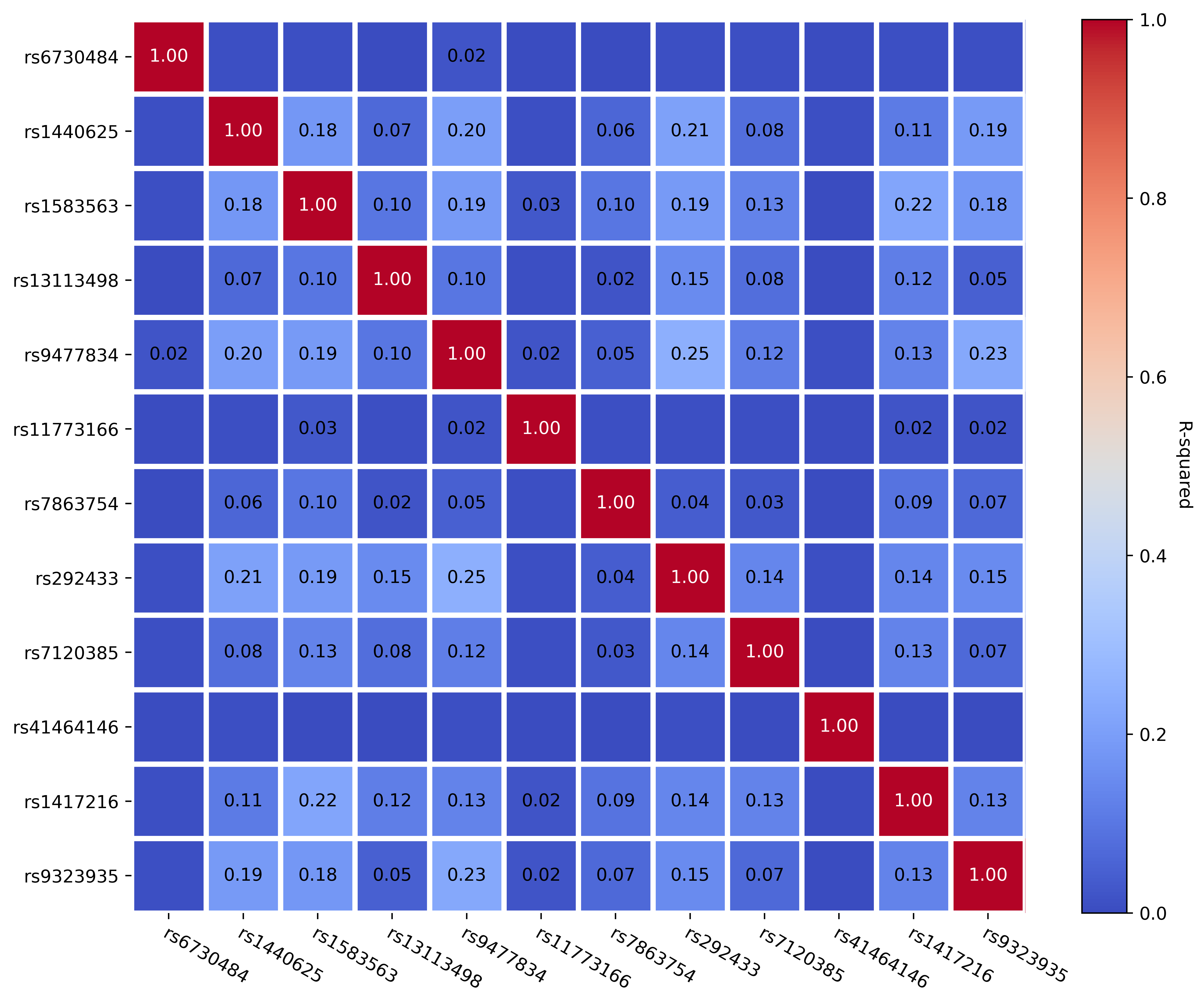


Pairwise R^2^ were calculated based on the SBCGS dataset.

**Figure S3** Hyperparameters tuning results of the logistic ridge regression model


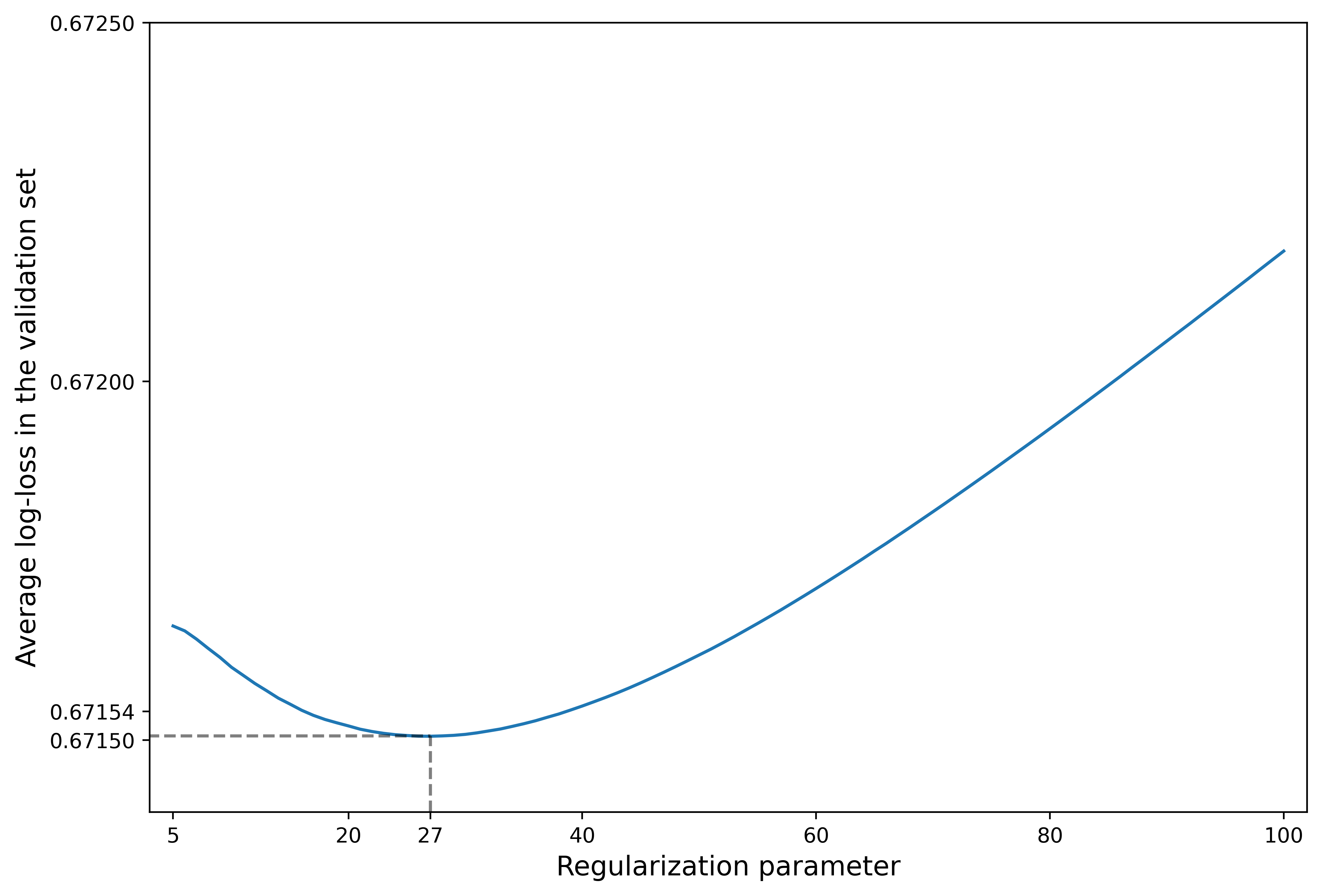


Hyperparameters tuning was conducted by applying 10-fold cross validation on the SBCGS dataset and using average log-loss as the main outcome; the optimal regularization parameter was 27.

**Figure S4** The structure of the ANN model used in the current study


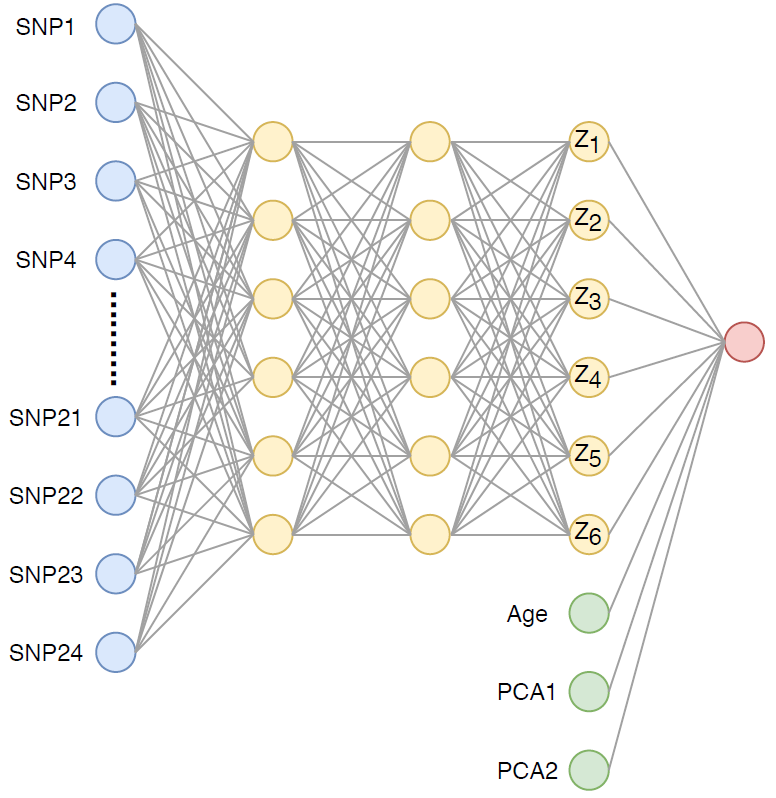


Bias neurons are not plotted on the graph; the blue dots represent the values of the 24-SNPs inputted into the model, the yellow dots represent the hidden neurons in the hidden layers, the green dots represent the covariates adjusted in the model, and the red dot represents the outcome variable; values of the hidden neurons in the third hidden layers ($Z_{1}-Z_{6}$) and their corresponding weighting parameters were used to calculate the PRS_ANN_: ${PRS}_{ANN}=\sum_{i=1}^{6} {\beta_{i}Z}_{i}$.
